# Green Hydrogels Composed of Sodium Mannuronate/Guluronate, Gelatin and Biointeractive Calcium Silicates/Dicalcium Phosphate Dihydrate Designed for Oral Bone Defects Regeneration

**DOI:** 10.3390/nano11123439

**Published:** 2021-12-18

**Authors:** Maria Giovanna Gandolfi, Fausto Zamparini, Sabrina Valente, Greta Parchi, Gianandrea Pasquinelli, Paola Taddei, Carlo Prati

**Affiliations:** 1 Laboratory of Green Biomaterials and Oral Pathology, School of Dentistry, DIBINEM, University of Bologna, 40125 Bologna, Italy; fausto.zamparini2@unibo.it (F.Z.); greta.parchi@studio.unibo.it (G.P.); 2 Endodontic Clinical Section, School of Dentistry, DIBINEM, University of Bologna, 40125 Bologna, Italy; carlo.prati@unibo.it; 3 Department of Experimental, Diagnostic and Specialty Medicine, DIMES, University of Bologna, 40138 Bologna, Italy; sabrina.valente2@unibo.it (S.V.); gianandr.pasquinelli@unibo.it (G.P.); 4 Subcellular Nephro-Vascular Diagnostic Program, Pathology Unit, IRCCS Azienda Ospedaliero-Universitaria di Bologna, 40138 Bologna, Italy; 5 Biochemistry Unit, DIBINEM, University of Bologna, 40126 Bologna, Italy; paola.taddei@unibo.it

**Keywords:** green biomaterials, natural polymers, algae-based hydrogels, seaweed-based hydrogels, bioactive minerals, calcium silicates (CaSi), dicalcium phosphate dihydrate (DCPD), natural polysaccarides, bone regeneration, oral bone defects, tissue engineering, regenerative medicine

## Abstract

Innovative green, eco-friendly, and biologically derived hydrogels for non-load bearing bone sites were conceived and produced. Natural polysaccharides (copolymers of sodium D-mannuronate and L-guluronate) with natural polypeptides (gelatin) and bioactive mineral fillers (calcium silicates CaSi and dicalcium phosphate dihydrate DCPD) were used to obtain eco-sustainable biomaterials for oral bone defects. Three PP-x:y formulations were prepared (PP-16:16, PP-33:22, and PP-31:31), where PP represents the polysaccharide/polypeptide matrix and x and y represent the weight % of CaSi and DCPD, respectively. Hydrogels were tested for their chemical-physical properties (calcium release and alkalizing activity in deionized water, porosity, solubility, water sorption, radiopacity), surface microchemistry and micromorphology, apatite nucleation in HBSS by ESEM-EDX, FT-Raman, and micro-Raman spectroscopies. The expression of vascular (*CD31*) and osteogenic (alkaline phosphatase *ALP* and osteocalcin *OCN*) markers by mesenchymal stem cells (MSCs) derived from human vascular walls, cultured in direct contact with hydrogels or with 10% of extracts was analysed. All mineral-filled hydrogels, in particular PP-31:31 and PP-33:22, released Calcium ions and alkalized the soaking water for three days. Calcium ion leakage was high at all the endpoints (3 h–28 d), while pH values were high at 3 h–3 d and then significantly decreased after seven days (*p* < 0.05). Porosity, solubility, and water sorption were higher for PP-31:31 (*p* < 0.05). The ESEM of fresh samples showed a compact structure with a few pores containing small mineral granules agglomerated in some areas (size 5–20 microns). PP-CTRL degraded after 1–2 weeks in HBSS. EDX spectroscopy revealed constitutional compounds and elements of the hydrogel (C, O, N, and S) and of the mineral powders (Ca, Si and P). After 28 days in HBSS, the mineral-filled hydrogels revealed a more porous structure, partially covered with a thicker mineral layer on PP-31:31. EDX analyses of the mineral coating showed Ca and P, and Raman revealed the presence of B-type carbonated apatite and calcite. MSCs cultured in contact with mineral-filled hydrogels revealed the expression of genes related to vascular (*CD31*) and osteogenic (mainly *OCN*) differentiation. Lower gene expression was found when cells were cultured with extracts added to the culture medium. The incorporation of biointeractive mineral powders in a green bio-derived algae-based matrix allowed to produce bioactive porous hydrogels able to release biologically relevant ions and create a suitable micro-environment for stem cells, resulting in interesting materials for bone regeneration and healing in oral bone defects.

## 1. Introduction

Tissue engineering is gaining increasing attention in oral bone surgery in relation to the need for new bone substitute materials.

Considering the high variability and complex architecture of bone defects in the oral and maxillofacial district, oral bone surgery requires tailorable, customizable, adaptable, and shapeable biomaterials for bone regeneration. These biomaterials should positively interact with mineralizing/remodeling cells, must be biocompatible and bioactive, easily insertable, and possibly requiring minimally invasive surgical procedures [1,2].

Different bone defects frequently occur in the oral and maxillofacial area. These defects may be caused by periradicular/periapical lesions and periapical abscesses, odontogenic cysts, peri-implantitis, periodontitis, bone trauma, bone atrophies and tumors, and are responsible for tooth loss, occlusal dysfunctions, and functional alterations.

A novel approach in tissue engineering is represented by the design of innovative green and biologically derived materials to combine good biological properties with eco-friendly productive processes.

In this context, the development of eco-sustainable materials produced by using biological/organic wastes (i.e., obtained from controlled fermentation of agro-wastes) or natural components represents an environmental-responsive approach of the new industrial science [3,4].

The use of organic wastes represents an ethical and valuable approach to reduce environmental pollution. Moreover, green chemistry does not entail the generation of toxic or harmful wastes during the production processes [3,4].

Polymers are widely used to design materials for tissue engineering and possess attractive properties for bone regeneration. Natural polymers, including peptides (gelatin and collagen), natural poly-esters (polyhydroxyalkanoates, poly(β-hydroxybutyrate and poly(β-hydroxybutyrate-co-β-hydroxyvalerate)) and polysaccharides (alginates i.e., mannuronate/guluronate-based copolymers, cellulose, chitin, hyaluronic acid, pectin and starch), have been used to prepare sponges [5], membranes [6], and recently porous scaffolds [7,8] as well for tissue engineering. Various polysaccharides form hydrogels through physical or chemical cross-linking, and many of them possess environmentally responsive properties. Hydrogel refers to cross-linked water-swollen polymer networks forming a porous structure with high water entrapment property and leaching capability.

Polysaccharide-based hydrogels have proven ideal for biomedical and pharmaceutical applications due to their intrinsic biocompatibility, degradability, environment sensitivity to pH, temperature, and possibility to incorporate specific biomolecules [9,10].

Porous hydrogels allow for the easier transport of mineralizing cells and nutrients into the biomaterials, ensuring a suitable environment for new bone tissue regeneration [11,12]. Natural polysaccharides are interesting components adopted as part of innovative approaches and strategies to fabricate biodegradable, biocompatible, and green matrices for regenerative medicine as they have demonstrated biocompatibility, no hydrophobic behavior, and biodegradation in non-toxic components.

Seaweed-derived (algae-based) natural polysaccharides, consisting of linear copolymers of D-mannuronic acid and L-guluronic acid units, showed the ability to retain water and gelling, forming biocompatible and stable hydrogels with adjustable porosity and low immunogenicity and biodegradability in physiological conditions. Gelation occurs in the presence of cross-linking divalent ions (as calcium and magnesium) and the mechanical properties of hydrogels increase with increasing concentrations of divalent ions [13]. In addition to the chemical cross-linking gelation, hydrogel preparation can be achieved using other ecologically sustainable non-toxic techniques, such as freeze casting [5,6] or phase separation and ultrasonic cavitation [14].

Mannuronic/guluronic acids-based polymers have been used to produce through the freeze casting method either 3D highly porous sponges/scaffolds with vasculogenic properties on human embryonic stem cells [5] or membranes having mechanical and physical properties adequate for skin tissue engineering [6].

Hydrogels, with their lack of native ligand sites suitable for mammalian cells attachment [13], showed in vivo uncontrolled degradation rates and low mechanical stiffness [15]. Hydrogels can be doped with magnesium phosphate or reinforced with polymer fibers to improve their mechanical properties and to provide a favorable substrate for cells [16] or can be combined with polypeptides, such as collagen and/or gelatin, to mimic the human extracellular matrix and provide sites for cell attachment and colonization [10].

Gelatin is a heterogeneous mixture of water-soluble peptides produced by the partial hydrolysis of collagen extracted from the skin, bones, and connective tissues of animals. Gelatin is biocompatible and biodegradable, exhibits low antigenicity, does not produce harmful by-products upon enzymatic degradation, and is available at low cost [10]. Gelatin has been recently used to produce 3D-printed scaffolds with high porosity, promising mechanical properties and no cytotoxic effects on human dermal fibroblasts [8].

The association of mannuronic/guluronic acids-based polymers and gelatin has been used in some biomedical applications, such as drug delivery systems [17], skin tissue engineering [18], and cartilage tissue engineering [4,19]. However, they do not appear suitable for applications in bone tissue engineering due to low mechanical properties, non-controllable resorbability, and difficulties inherent to reaching high porosity values [20].

Hydraulic calcium silicates (CaSi) demonstrated to be effective fillers in bone regeneration procedures in dentistry [21] in relation to their biointeractivity [22,23,24], apatite forming ability [22,23,25,26,27], biocompatibility, and capability to induce the differentiation of several mineralizing cells, such as human bone marrow stromal cells [28,29], orofacial bone mesenchymal stem cells [30], cementoblasts [31], and oral derived periapical cyst mesenchymal stem cells [32].

The combination of calcium phosphates such as dicalcium phosphate dihydrate (DCPD) to CaSi materials was demonstrated to enhance their biological properties [30,33,34], and apatite-forming ability [33,35,36].

The novelty of the present study was to design and produce seaweed-based green mineral-filled hydrogels, constituted of copolymers of sodium D-mannuronate and L-guluronate units, gelatin, and nanoparticles of CaSi and DCPD, conceived for the regeneration of non-load bearing oral bone defects and to evaluate their chemical-physical and biological (osteogenic and angiogenic commitment of stem cells) properties. Similar formulations have never been reported.

## 2. Materials and Methods

### 2.1. Mineral-Filled Hydrogels Preparation

The hydrogels were designed and prepared in the Laboratory of Green Biomaterials at the Dental School of the University of Bologna.

Poly(sodium D-mannuronate-co-L-guluronate) powder (Sigma Aldrich, St. Louis, MO, USA), gelatin from bovine skin powder (Sigma Aldrich, St. Louis, MO, USA), and calcium chloride (Sigma Aldrich, St. Louis, MO, USA) were used.

Dicalcium phosphate dihydrate (DCPD; CaHPO_4_·2H_2_O, Sigma-Aldrich, Steinheim, Germany), wollastonite, and CaSi powder (Aalborg, Denmark)—composed of dicalcium silicate, tricalcium silicate, tricalcium aluminate, and calcium sulfate and prepared by melt-quenching technique followed by grinding using agate ball milling procedures (particle size less than 5 microns) [22,35,37]—were used as bioactive biointeractive mineral fillers.

Poly(sodium D-mannuronate-co-L-guluronate) and gelatin components were present in a 1:1 weight ratio and formed the organic hydrogel phase Polysaccharide/Polypeptide (PP).

PP was filled with various amounts of mineral powders and different PP-x:y formulations were prepared, where x and y represent the weight % of CaSi and DCPD, respectively. Three formulations were studied: PP-16:16, PP-33:22, PP-31:31.

**Figure 1:**
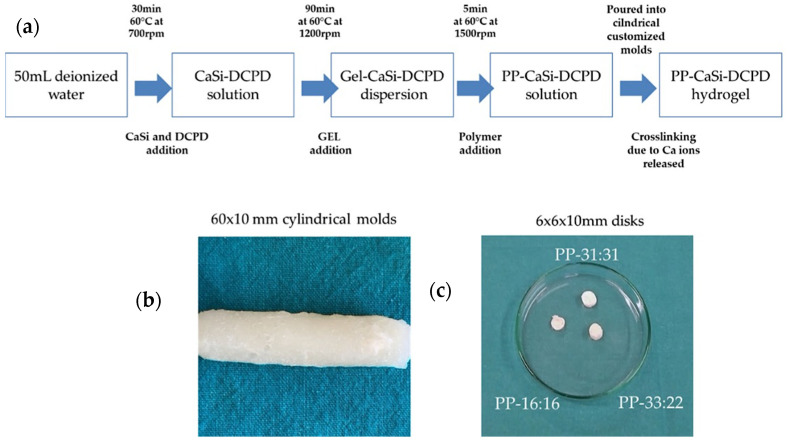
(**a**) Preparation of mineral-filled hydrogels. Calcium ions released into the solution from CaSi and DCPD minerals were used for hydrogel cross-linking. (**b**) Mineral-filled hydrogel removed from the cylindrical mold. (**c**) Hydrogel disks of the prepared formulations.

Bioactive mineral powders were added in 50 mL of demineralized water and kept at 60 °C and 700 rpm for 30 min under vigorous stirring, to achieve a homogeneous dispersion. Then, gelatin (polypeptide) powder was slowly added to the dispersion and maintained at 60 °C and 1200 rpm for 90 min. Finally, the copolymer of D-mannuronate/L-guluronate sodium salts (polysaccharide) was added to the dispersion and vigorously stirred at 60 °C and 1500 rpm for 5 min (Figure 1a). The slurry mixture was then poured on paraffin-covered Petri dishes (diameter 60 mm, height 10 mm), or a polydimethylsiloxane cylindrical mold (diameter 10 mm, height 60 mm) and cooled at 0–4 °C for at least 2 days (Figure 1b,c). The hydrogel solidification through chemical cross-linking was obtained by the calcium ions released from CaSi and DCPD mineral fillers.

Hydrogel without mineral fillers (used as control, PP-CTRL) was prepared as follows: 1g CaCl_2_ was dissolved in 50 mL of demineralized water and kept at 60 °C and 700 rpm for 20 min under vigorous stirring to achieve a homogeneous dispersion. Gelatin powder was then added and stirred at 60 °C and 1000 rpm for 60 min. Finally, the copolymer of D-mannuronate/L-guluronate sodium salts powder was added to the dispersion (gelatin to copolymer weight ratio 1:1) and vigorously stirred at 1500 rpm. The slurry mixture was poured inside paraffin-covered Petri dishes (diameter 60 mm, height 10 mm) or into polydimethylsiloxane-based cylindrical molds (diameter 10 mm, height 60 mm) and cooled at 0–4 °C for at least 2 days.

### 2.2. Calcium Release and Alkalizing Activity (pH of Soaking Water)

Cylindrical molds (10 ± 0.1 mm diameter and 10 ± 0.1 mm thick; *n* = 10 for each composition) were immersed in 10 mL of deionized water inside polypropylene-sealed containers and stored at 37 °C. The soaking water was collected and replaced at six-time endpoints (3 h and 1, 3, 7, 14, and 28 days). The collected water was analyzed for pH and calcium ions using a potentiometric method under magnetic stirring at room temperature (25 °C). The pH was measured using a selective temperature-compensated electrode (Sen Tix Sur WTW, Weilheim, Germany) connected to a multi-parameter laboratory meter (inoLab 750 WTW, Weilheim, Germany) previously calibrated with standard solutions. The amount of calcium ions was measured using a calcium probe (calcium ion electrode, Eutech instruments Pte Ltd., Singapore City, Singapore) after the addition of 2% of ionic strength adjuster (ISA) 4 mol/L KCl (WTW, Weilheim, Germany).

### 2.3. Solubility, Porosity, Water Sorption

Cubic samples (10 ± 0.1 mm long × 10 ± 0.1 mm wide × 10 ± 0.1 mm thick; *n* = 8 for each composition) were weighed to determine the initial mass (I) and the mass immersed in 20 mL of distilled water at 37 °C for 24 h (S). Then, the specimens were removed from the water, the excess water from the surface of each sample was removed using a moistened filter paper (20 mL of distilled water dropped on a 9 cm wide 12.5-cm-long glass plate covered by a filter paper), and the saturated mass (M) was recorded.

Finally, the samples were dried at 37 °C until the weight was stable, and the final dry mass (D) was recorded.

Open pores volume (V_OP_ = M − D, in cm^3^), impervious portion volume (V_IP_ = D − S, in cm^3^), and apparent porosity (P = [(M − D)/V] × 100) were calculated following the Archimedes principle (and ASTM C266-88) [24,33,38].

Water sorption (WS = [(M − D)/D] × 100) and solubility (S = [(I − D)/D] × 100) were calculated as a percentage of the original weight. Each weight measurement was repeated three times using an analytical balance (Bel Engineering series M, Monza, Italy) and determined to the nearest 0.001 g. Mean values of the measures were reported.

### 2.4. Setting Time

Cylindrical samples (10 ± 0.1 mm diameter and 10 ± 0.1mm thick; *n* = 3 for each composition) were placed at 37 °C and 95% relative humidity. The initial setting time was measured by evaluating the absence of indentation caused by a Gilmore needle (ASTM C 226-07 Standard test method for time of setting of cement paste by Gillmore needles) [22,24,33,38] at room temperature (25 °C). The Gilmore needle to evaluate the initial setting time weighed 113.4 g and was 2.12 mm in diameter.

### 2.5. Radiopacity

Cylindrical samples (10.0 mm diameter, 1.0 mm thick; *n* = 3 per group) were radiographed using a radiographic unit with a reference aluminum step wedge (60 mm long, 10 mm wide thickness varying from 2 to 6 mm in 1 mm-increments) following ISO 9917-2007 [24,38,39]. The target-film distance was approximately 30 cm with the sample at 3 cm from the surface of the radiographic tube, 0.13 s exposure at 70 KVp, and 8 mA. The film was processed and scanned. The radiographic density (color intensity) data were converted into aluminum step-wedge equivalent thickness (mmAl) using the software Image J (NIH software, Bethesda, MD, USA) [24,38,39].

### 2.6. Surface Micromorphology and Apatite Nucleation in Hank Balanced Salt Solution

The apatite forming ability was evaluated following ISO 23317 (implants for surgery—in vitro evaluation for apatite-forming ability of implant materials). Hank Balanced Salt Solution (HBSS, Cambrex Bioscience, Verviers, Belgium) having a composition of inorganic ions similar to human blood plasma was used as simulated body fluid [23,24,33,40,41]. HBSS composition was: K^+^ 5.8 mM, Ca^2+^ 1.27 mM, HCO_3_^−^ 4.17 mM, SO_4_^2−^ 0.81 mM, Mg^2+^ 0.81 mM, Na^+^ 141.6 mM, H_2_PO_4_^−^ 0.44 mM, HPO_4_^2−^ 0.336 mM, and Cl^−^ 144.7 mM.

Set fresh samples and samples aged 28 days in HBSS were analysed by ESEM-EDX, FT-Raman, and micro-Raman.

An environmental scanning electron microscope (ESEM, Zeiss EVO 50; Carl Zeiss, Oberkochen, Germany) connected to a secondary electron detector for energy dispersive X-ray analysis (EDX; Oxford INCA 350 EDS, Abingdon, UK) using computer-controlled software (Inca Energy Version 18, Abingdon, UK) was used. Specimens were placed directly on the ESEM stub and examined uncoated in wet conditions at low vacuum (100 Pascal). EDX microchemical analysis was carried out in random areas of 50 × 50 µm to evaluate the relative element content. Elemental microanalysis (weight % and atomic %) with the ZAF correction method was performed in full frame to analyze entire areas [23,24,33,40,41].

Spectroscopic vibrational techniques (FT-Raman and micro-Raman) were used to characterize the mineral fillers, polymeric matrices, and mineral-filled hydrogels before (i.e., fresh samples) and after apatite nucleation tests.

FT-Raman spectra were recorded by using a Bruker MultiRam FT-Raman spectrometer equipped with a cooled Ge-diode detector (Bruker Optik GmbH, Ettlingen, Germany). The excitation source was a Nd^3+^-YAG laser (1064 nm) in the backscattering (180°) configuration. The focused laser beam diameter was about 100 µm, the spectral resolution 4 cm^−1^, and the laser power for the sample was about 120 mW. Three-five Raman spectra were non-destructively recorded on three-five different positions of each composite sample and averaged.

Micro-Raman spectra were obtained by using an NRS-2000C Jasco spectrometer (Jasco Inc., Easton, MD, USA) with a microscope of 100× magnification. All the spectra were recorded in back-scattering conditions with 5 cm^−1^ spectral resolution by using the 532 nm green diode-pumped solid-state laser driver (RgBLase LLC, Fremont, CA, USA) with a power of about 20 mW. A 160 K cooled digital charge-coupled device (Spec-10: 100B, Roper Scientific Inc., Sarasota, FL, USA) was used as a detector. Due to the poor quality of the micro-Raman spectra of the fresh samples, this technique was only used to investigate the presence of a mineral deposition on the hydrogels, in addition to FT-Raman spectroscopy. This technique was also used to characterize the fresh samples and to disclose possible interactions between the phases through the comparative analysis of the spectra.

### 2.7. Cell Tests

#### 2.7.1. Hydrogel Disks Sterilization

Cylindrical samples (10.0 mm diameter, 1.0 mm thick) were immersed in 250 mL of 70% ethanol solution for 20 min, then rinsed for 3 consecutive times with 5 mL of distilled water using a syringe [42].

#### 2.7.2. Cells Test: Direct Contact and Extract Test

Human mesenchymal stem cells (MSCs) derived from vascular wall were harvested in accordance with Local Ethics Committee Approval (protocol APP-13-01). The isolation process and stemness properties are detailed elsewhere [43]. Human MSCs were plated at the density of 5.5 × 10^4^ cells/well in 12-well plates and cultured in Dulbecco’s Modified Eagle’s Medium (DMEM, Euroclone, Milan, Italy) with 10% Fetal Bovine Serum (FBS, Euroclone, Milan, Italy) in an incubator at 37 °C and 5% CO_2_ to allow cell adhesion and monolayer formation.

Hydrogels disks (PP-16:16, PP-33:22, PP-31:31) were placed into the wells in contact with MSCs and cultured for 3, 7, and 14 days. The culture medium was changed every 3 days. MSCs cultured without hydrogels were used as control. These experiments were used to evaluate the effects of hydrogels on vasculogenic and osteogenic MSCs differentiation.

Additional experiments using the extracts from each hydrogel formulation were performed to evaluate the biocompatibility (cell mortality) and MSCs commitment (expression of vasculogenic and osteogenic markers). ISO 10993-5:2009 (clause 4.2.3.2: Extraction conditions) was used to prepare the extracts by using a 37 °C extraction temperature instead of 50 °C to simulate the clinical conditions. Extracts were obtained by immersion of hydrogel disks (10.0 mm diameter, 1.0 mm thick) into 5 mL of sterile water for 72 h followed by filtration before their addition to culture medium. MSCs were incubated for 3 days in DMEM with 10% FBS and 10% of extract from each hydrogel formulation, and cell mortality was assessed in each experimental condition. Briefly, MSCs were detached, centrifuged, and manually counted. The mortality was calculated as the ratio between the death cells number and the total cells number × 100, and values were expressed as percentages.

#### 2.7.3. *CD31*, *ALP* and *OCN* Gene Expression

Total RNA was extracted from MSCs placed in contact with different hydrogel formulations using PureZOL (Bio-Rad, Hercules, CA, USA) according to the manufacturer’s instructions. Reverse transcription was performed from 0.25 ng RNA through iScript cDNA Synthesis Kit (Bio-Rad, Hercules, CA, USA). Real-time PCR was carried out using SsoAdvanced™ Universal SYBR^®^ Green Supermix (Bio-Rad Hercules, CA, USA) and amplified through CFX Connect™ Real-Time PCR Detection System (Bio-Rad Hercules, CA, USA). The forward and reverse primers sequences (Sigma-Merck, Milan, Italy) are listed in Table 1. Glyceraldehyde 3-phosphate dehydrogenase (*GAPDH*) was used as housekeeping gene. The expression of target genes was normalized on *GAPDH* and analyzed using the 2^^−ΔΔCt^ relative quantification methods. Results were expressed as fold changes relative to the CTRL group.

The expression of *CD31* (a specific marker of vascular differentiation), Alkaline phosphatase (*ALP*), and Osteocalcin (*OCN*) (both markers of osteogenic differentiation) were analyzed.

### 2.8. Statistical Analysis

Statistical analysis of the chemical physical tests was performed using Sigmaplot 12 (Systat, Chicago, IL, USA). Calcium release and alkalizing activity were analyzed using two-way ANOVA followed by RM Student–Newman–Keuls test (*p* < 0.05). Different letters represent statistically significant differences (*p* < 0.05) in the same line (capital letters) or in the same column (small letters). A one-way ANOVA followed by RM Student–Newman–Keuls test (*p* < 0.05) was used to analyse statistically significant differences of the materials for solubility, porosity, water absorption, radiopacity, and setting time.

Statistical analysis of cell tests were carried out using GraphPad Prism software (San Diego, Ca, USA). The differences between experimental groups were evaluated using two-way ANOVA followed by Dunnett’s multiple comparison test. Results from three independent experiments are reported as mean ± standard deviation (SD). *p* values < 0.05 were considered statistically significant.

## 3. Results

### 3.1. Calcium Release and Alkalizing Activity

The release of calcium ions and the alkalizing activity (pH of soaking solution) of the mineral-filled hydrogels after their immersion in deionized water up to 28 days have been analysed to study the biointeractive properties of the designed biomaterials.

Calcium release values of the hydrogels are reported in Table 2. High calcium ions release values were observed from the first hours following immersion (3 h), with a significant increase up to the first three days. During this period, PP-31:31 revealed the highest values after one day, followed by PP-33:22 and PP-16:16, significantly different from PP-CTRL (*p* < 0.05).

After three days, PP-16:16 and PP-31:31 revealed a general decrease of calcium ions leaching, which was similar on both formulations. The mean values after 28 days reached 32.30 ± 13.18 and 27.73 ± 7.01, respectively.

PP-33:22 showed a constant calcium ion release up to 28 days, significantly higher than PP-16:16. PP-33:22 also revealed the highest cumulative calcium ions release (mean value was 253.15 ± 40.64).

Hydrogels without fillers revealed a slight calcium ion release after the first 24 h, which decreased but remained constant at 28 days.

The pH values are reported in Table 3. Alkaline pH values of soaking water were induced for all mineral-filled formulations. In particular, PP-33:22 and PP-31:31 revealed high pH values (over 9) during the first 24 h. After three days, the pH values drop to approximately 8.5, and slight alkaline values (over 8) were maintained for all the tested endpoints.

Samples with no mineral fillers showed neutral values (between 7.40 and 7.80) of soaking solution at all the endpoints, with a slight decrease only after three days.

### 3.2. Solubility, Porosity, Water Absorption

The evaluation of the solubility, porosity, and water sorption of the mineral-filled hydrogels was performed to evaluate their physical properties after immersion in water. The mean values are reported in Table 4.

Mineral-filled hydrogels revealed significant variations on the basis of the filler content. Samples with a lower amount of CaSi (PP-16:16) revealed values close to PP-CTRL, while the material with the highest amount of CaSi-DCPD (i.e., PP-31:31) revealed the highest solubility (S), porosity (P), and water sorption (WS) values (*p* < 0.05). Samples with no mineral fillers showed low porosity values (mean values were 36.47 ± 0.91) when compared to mineral-filled samples. The open pore volume V_OP_ and P increased while the volume of impervious portion V_IP_ decreased with the increment of the amount of the mineral fillers. Similarly, the solubility (S) and water sorption (WS) increased with increasing the mineral fillers content. The water sorption increase (WS) was directly related to the increase of material solubility (S). In a similar way, higher porosity values (P) were directly related to the open pore volume (V_OP_) and reduced volume of impervious portion (V_IP_).

### 3.3. Radiopacity

When biomaterials need to be monitored inside the bone defect for specific clinical reasons as in oral surgery, the radiopacity test provided a value of their detectability (Table 5). All the formulations revealed low radiopacity values.

### 3.4. Setting Time

Setting time tests have been performed to investigate the time required to obtain a stable biomaterial after the preparation processes.

All the mineral-filled materials showed an initial setting time of 96 h, while PP-CTRL was unable to completely set even after 144 h (Table 6).

### 3.5. Surface Micromorphology and Apatite Nucleation in HBSS

Surface micromorphology and Apatite nucleation in HBSS were performed to predict the in vivo modifications of mineral-filled hydrogels. ESEM-EDX and FT-Raman analyses allowed to detect the nucleation of amorphous calcium phosphates, precursors of biological apatites.

#### 3.5.1. ESEM-EDX

##### PP-16:16

**Figure 2:**
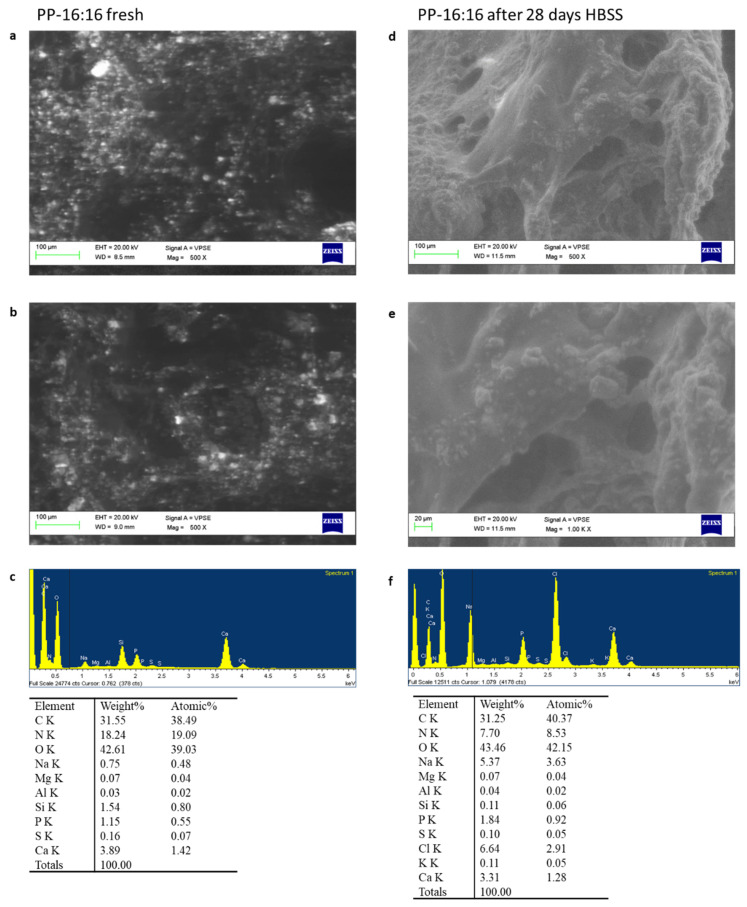
ESEM-EDX analysis at 500× (**a**) and 1000× (**b**) of PP-16:16 formulation before and after immersion in HBSS. The analysis on fresh samples revealed a regular surface with low porosities. EDX revealed the constitutional elements of the mineral filled hydrogels (**c**). After 28 days immersion in HBSS, ESEM at 500× (**d**) and 1000× (**e**) revealed the presence of larger circular and elliptic pores (20–120 µm range) and the formation of a mineral layer. EDX analysis evidenced an increase of P (from HBSS) and a decrease of Si (from the mineral powder) (**f**).

ESEM images at 500× magnifications revealed a flat surface with few irregular pores (Figure 2a). Electron dense granules (mean size 5–15 µm) attributable to CaSi and DCPD powders were well distributed on the entire material surface. This structure was well evident at 1000× magnification (Figure 2b). EDX revealed constitutive elements of the natural polymers, namely carbon (C), oxygen (O), and nitrogen (N), and of the mineral fillers, namely calcium (Ca), silicon (Si), and phosphorous (P) (Figure 2c).

After 28 days HBSS immersion, surface morphology at 500× and 1000× magnification showed a higher porosity, with circular and elliptic pores, ranging from 20 to 120 µm (Figure 2d,e). EDX revealed constitutive elements of the natural polymers, namely C, O, and N, sodium (Na) and of the mineral phase, namely Ca and P. A marked Si decrease was detected. Traces of chlorine (Cl) from HBSS solution were also detected (Figure 2f).

##### PP-33:22

**Figure 3:**
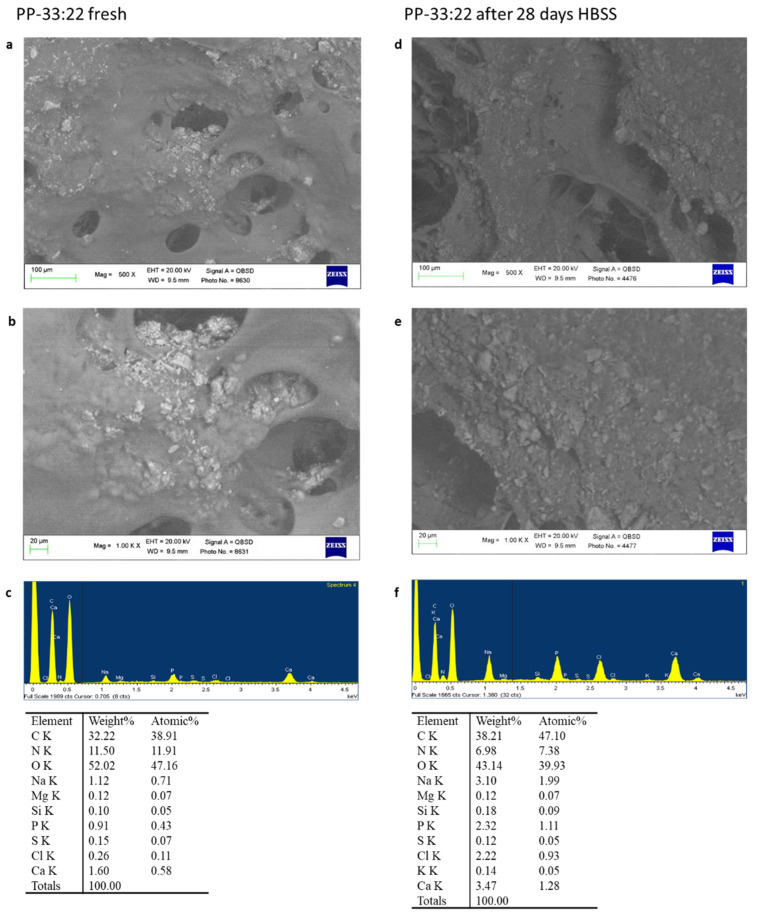
ESEM-EDX analysis at 500× (**a**) and 1000× (**b**) of PP-33:22 formulation before and after immersion in HBSS. Fresh samples showed some circular pores on their surface, with sparse light electron dense granules, attributable to the mineral nano-powders. In some areas these powders were conglomerated and formed larger granules. EDX revealed the structural elements of the formulation (**c**). ESEM-EDX analysis at 500× (**d**) and at 1000× (**e**) after 28 days immersion in HBSS. Large pores were detected on the hydrogel surface, with the exposure of more internal porosities. EDX revealed the increase of Ca and P, attributable to the mineral layer deposition (**f**).

ESEM images at 500× magnifications revealed a less regular area with several irregular porous structures (Figure 3a). Circular and elliptic pores, ranging from 20 to 80 µm were detected. Their internal portion revealed numerous electron dense granules. At high magnification (1000×), some pores completely covered by the electron dense granules may be identified (Figure 3b). EDX micro-analysis revealed the constitutional elements of the hydrogel, namely C, O, N, Na, and of the mineral powders, namely Ca, P, and Si (Figure 3c).

After 28 days immersion in HBSS, the hydrogels revealed large elliptic-shaped pores and the presence of an irregular layer on the external surface, as evident in Figure 3d,e. Degradation of the hydrogel surface may be detected, with additional exposures of hydrogel internal porosities and small electron dense granules. The size of porosities varied from 80 to 200 µm. EDX revealed an increase of P and Ca, the constitutional elements of polymer hydrogel (C, O, N, Na) and from HBSS (Na, K, and Cl) (Figure 3f).

##### PP-31:31

**Figure 4:**
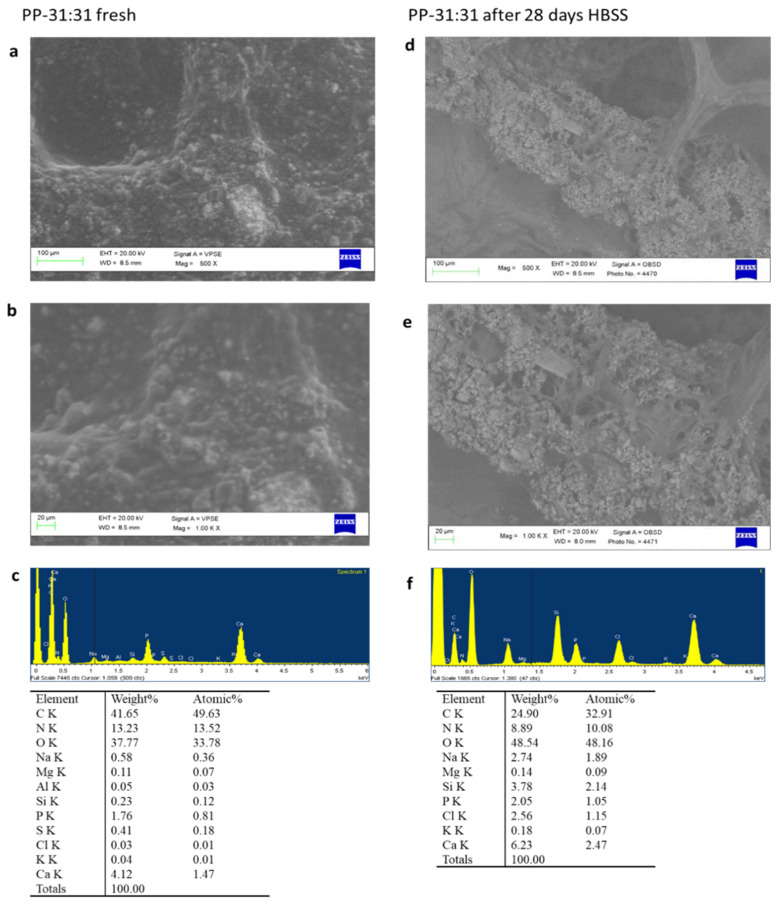
ESEM-EDX analysis at 500× (**a**) and 1000× (**b**) of PP-31:31 formulation before and after immersion in HBSS. An irregular surface is showed with the large presence of light electron dense granules of mineral powders. EDX analysis showed the constitutional elements of the formulation (**c**). ESEM-EDX analyses at 500× (**d**) and 1000× (**e**) of 28d-aged samples showed a markedly more porous surface with the exposition of more mineral granules (attributable to the Si increase) and the formation of a mineral layer, as revealed by the increase of Ca and P (**f**).

ESEM images at 500× and at 1000× magnifications revealed an irregular surface with limited circular pores, the sizes ranging from 30 to 80 µm (Figure 4a,b). Electron dense granules, attributable to CaSi and DCPD fillers, were well distributed on the entire material surface. Some micropores may be identified at higher magnifications, although completely covered by the mineral layer. EDX analysis revealed the constitutional elements of natural polymers (C, O, N, and Na) and mineral powders (Ca, P, Si, and S) (Figure 4c).

After 28 days HBSS immersion, ESEM at 500× and 1000× revealed a porous structure with a partially degraded matrix covered by an electron dense layer (Figure 4d,e). This layer was not regularly distributed on the entire material surface. Pores were mostly circular and elliptical-shaped. EDX analysis revealed the presence of the constitutional elements of the natural polymers (C, O, N, and Na), a moderate increase of P, Ca, and Si, due to the exposure of additional CaSi granules from the material (Figure 4f).

#### 3.5.2. FT-Raman and Micro-Raman Analyses

Figure S1 shows the FT-Raman spectrum of fresh PP-CTRL hydrogel. The comparison with the spectra of gelatin and poly(sodium D-mannuronate-co-L-guluronate) powders allowed the assignments of the bands to the two components, as also reported in Table S1. The shifts of several bands as well as the changes in bandwidths are not surprising, due to the presence of water and hydrogel formation. The COO^−^ symmetric stretching band was observed at 1414 cm^−1^ in sodium alginate, in agreement with the literature [44]. This mode shifted to 1419 cm^−1^ in the CTRL hydrogel.

**Figure 5:**
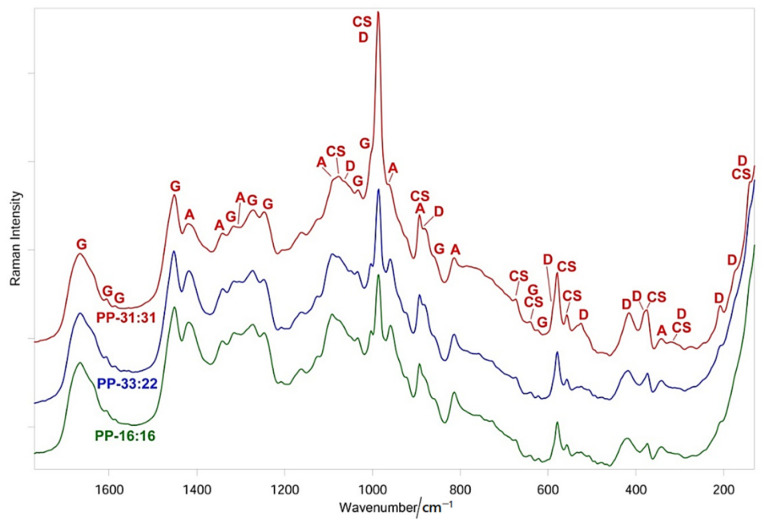
Average FT-Raman spectra of fresh PP-16:16, PP-33:22 and PP-31:31. The bands prevalently assignable to gelatin (G), poly(sodium D-mannuronate-co-L-guluronate) (A), DCPD (D) and CaSi (CS) are indicated. The spectra are normalized to the intensity of the Amide I band of gelatin at about 1666 cm^−1^.

Figure 5 shows the FT-Raman spectra of fresh PP-16:16, PP-33:22, and PP-31:31. Band assignments were given by comparison with the spectra of fresh PP-CTRL hydrogel, DCDP, and CaSi powders (Figure S2 and Table S2). As can be easily seen from the spectra, the relative intensity of the bands assignable to the inorganic components increases as their contents increase. An analogous behavior was observed for the bands of the organic hydrogel, which appear more prominent in the spectra of the samples that contain the lowest amounts of inorganic fillers. The presence of the latter appears to affect the organic hydrogel, as observable from the changes in the Amide I (1666 cm^−1^ band, Figure S3) and Amide III ranges (1272–1247 cm^−1^ bands) of gelatin and in the COO^−^ symmetric stretching region (which appeared further shifted to 1421 cm^−1^). As detailed in Table S2, several bands, assignable to both inorganic and organic components, underwent wavenumber shifts upon formation of the mineral-filled hydrogels.

**Figure 6:**
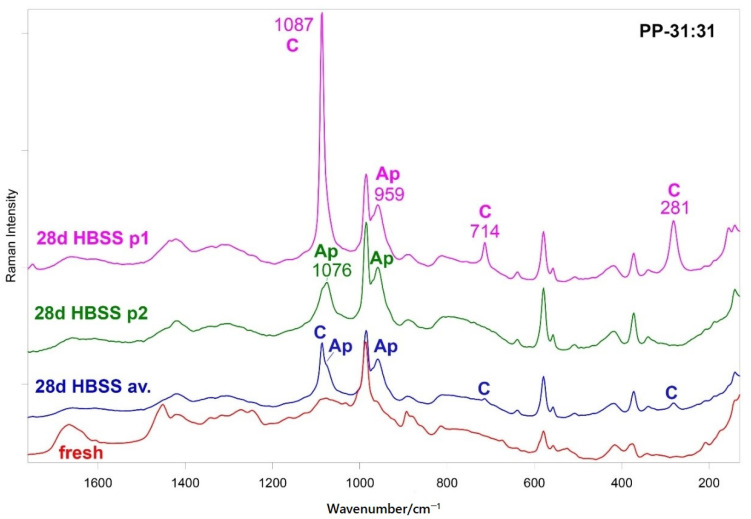
Average FT-Raman spectra of fresh PP-31:31 and after ageing for 28 days in HBSS; the single spectra recorded on two different points of the aged specimen are shown as well (p1 and p2). The bands prevalently assignable to calcite (C) and B-type carbonated apatite (Ap) are indicated. The spectra are normalized to the intensity of the band at 987 cm^−1^ assignable to the inorganic fillers.

Figure 6 shows the FT-Raman spectra of fresh PP-31:31 and of the same hydrogel aged for 28 days in HBSS. The average spectrum of the latter sample is shown together with the single spectra recorded on two different points of the same specimen. The spectra were normalized to the intensity of the band at 987 cm^−1^ assignable to the inorganic fillers. The average spectrum shows the bands of calcite at 1087, 714 and 281 cm^−1^ [45] together with the PO_4_^3−^ stretching of calcium phosphates at 959 cm^−1^. The single spectrum recorded in p1 was dominated by the bands of calcite, while that recorded in p2 allowed to clarify that the calcium phosphate formed was a B-type carbonated apatite, revealed through the CO_3_^2−^ stretching band at 1076 cm^−1^ [46].

**Figure 7:**
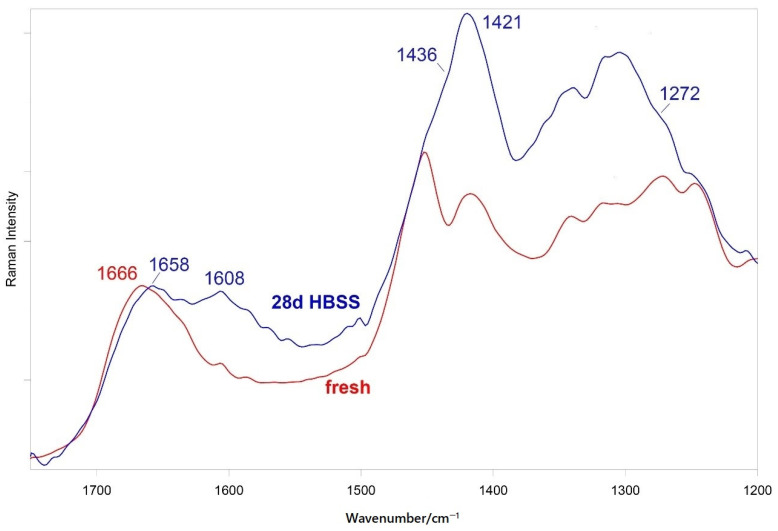
Average 1750-1200 cm^−1^ FT-Raman spectra of fresh PP-31:31 and after ageing for 28 days in HBSS. The spectra are normalized to the intensity of the Amide I band of gelatin at about 1666 cm^−1^.

The micro-Raman analysis confirmed these findings. Figure S4 shows the micro-Raman spectra recorded on two different points of the aged PP-31:31 hydrogel. The spectrum recorded in p1 confirms the presence of calcite, while that recorded in p2 showed the bands of B-type carbonated apatite, together with those of the hydrogel. In the average and single spectra of the aged PP-31:31 hydrogel reported in Figure 6, the bands of the hydrogel were still detected, although weakened with respect to the fresh sample. To gain more insights into the organic components, Figure 7 shows the average 1750–1200 cm^−1^ FT-Raman spectra of the fresh PP-31:31 hydrogels before and after the in vitro bioactivity test, normalized to the intensity of the Amide I band of gelatin. As can be easily observed, the band assigned to the COO^−^ symmetric stretching at 1420 cm^−1^ appeared more prominent than before ageing and became the most intense in this spectral range. A shoulder at 1436 cm^−1^ assignable to calcium alginate was detected [47].

At the same time, the band at 1608 cm^−1^ increased in relative intensity due to the contribution of the COO^−^ asymmetric stretching.

As detailed in Table S3, several bands of the hydrogels underwent shifts upon ageing and some bands significantly decreased their relative intensity. In particular, the Amide I of gelatin shifted from 1666 to 1658 cm^−1^ and the 1272 cm^−1^ Amide III component strengthened.

The FT-Raman spectra of the aged PP-16:16 and PP-33:22 hydrogels did not provide useful information since the fluorescence of these samples almost completely masks the Raman spectrum (Figures S5 and S6). The micro-Raman spectra did not show either the bands of calcite or B-type carbonated apatite.

### 3.6. Cell Tests

Cell tests were performed to analyse the effects of the mineral-filled formulations on MSCs. In particular, MSCs mortality and the expression of pro-angiogenic and pro-osteogenic markers were evaluated.

#### 3.6.1. Biocompatibility

**Figure 8:**
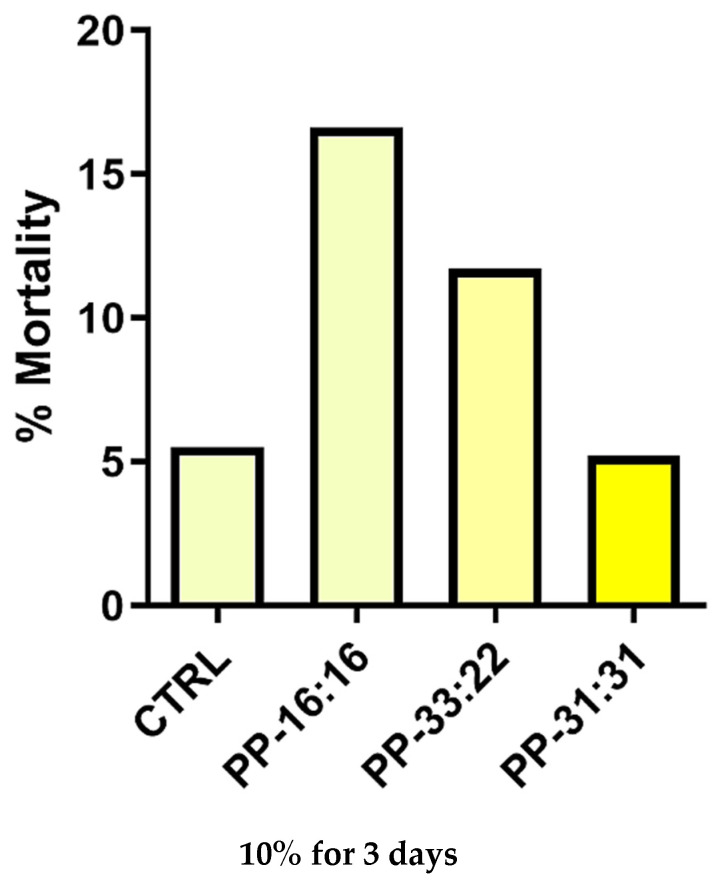
Mortality percentage of MSCs cultured with the addition of 10% extracts from mineral-filled formulation at the growth medium for 3 days. PP-31:31 showed similar mortality values when compared to CTRL (control MSCs cells cultured in absence of extracts). Values are expressed as percentages.

The MSCs mortality cultured in medium with the addition of 10% extracts from mineral-filled hydrogels is reported in Figure 8. After three days, the mortality of MSCs cultured with extracts from the formulation with the highest amount of CaSi and DCDP (PP-31:31) was similar to the CTRL (control MSCs grown without extracts; 5.5% mortality). PP-16:16 and PP-33:22 showed a higher mortality rate (16.6 % and 11.7 % respectively).

#### 3.6.2. Vascular Differentiation (*CD31* Gene Expression) of MSCs in Contact with the Mineral-Filled Hydrogels

**Figure 9:**
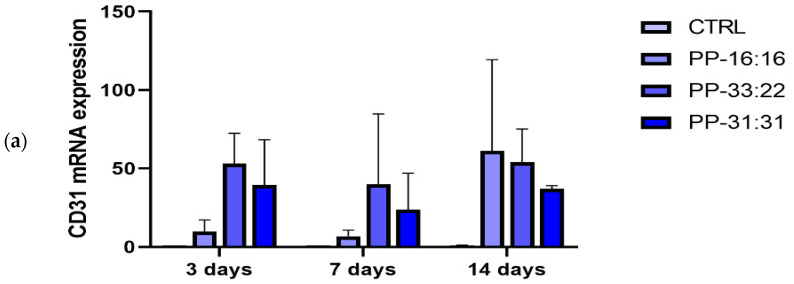

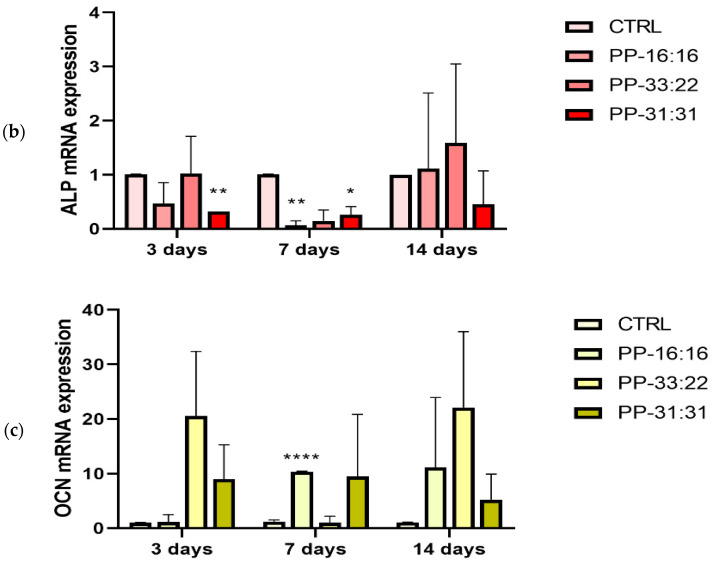
Gene expression of *CD31* (**a**), *ALP* (**b**) and *OCN* (**c**) analyzed by Real Time PCR in MSCs cultured in contact with all the mineral-filled hydrogels/formulations (PP-16:16, PP-33:22, PP-31:31). Results are reported as fold changes relative to CTRL (MSCs cultured without hydrogels/formulations) and expressed as mean ± SD of three independent experiments. *ALP*: alkaline phosphatase. *OCN*: osteocalcin. Asterisks mean statistically significant differences; 3 days: ** *p* = 0.0070 vs. CTRL; 7 days: ** *p* = 0.0053 and * *p* = 0.0265 vs. CTRL; **** *p* <0.0001 vs. CTRL. Bars indicate the Standard Deviation.

The mRNA expression of *CD31* (endothelial vascular differentiation marker) increased in MSCs cultured in contact with all the mineral-filled hydrogels (Figure 9a). At 3 days, the formulations with the highest amounts of CaSi and DCPD (PP-33:22 and PP-31:31) induced a marked up-regulation (over 40-fold increase) of *CD31* in comparison to CTRL. These values decreased at seven days, then increased to higher values after 14 days.

PP-16:16 showed a 8.7- and 5.7-fold increase of *CD31* gene expression at three and seven days, respectively, compared with the CTRL (MSC control cells). At 14 days, the mRNA expression reached similar values to PP-33:22 and PP-31:31.

#### 3.6.3. Osteogenic Differentiation (*ALP* and *OCN* Gene Expression) of MSCs in Contact with the Mineral-Filled Hydrogels

During the observational time (3–14 days), the mRNA expression of *ALP* (marker of osteogenic differentiation) was down regulated in MSCs cultured on mineral-filled hydrogels in comparison to the CTRL (MSC control cells).

PP-16:16, the formulation with the lowest percentage of CaSi and DCPD, showed a decrease of *ALP* expression. At 14 days, an increase similar to CTRL was observed. Similarly, PP-31:31, the highly filled formulation, significantly reduced *ALP* gene expression. MSCs cultured close to PP-33:22 showed a 0.58-fold increase of the mRNA expression of *ALP* at 14 days (Figure 9b).

The mRNA expression of *OCN* by cells cultured on mineral-filled hydrogels (late marker of osteogenic differentiation) was higher than CTRL (control cells) in almost all the cases (Figure 9c). MSCs cultured in contact with PP-16:16 showed a general increase of *OCN* expression at seven and 14 days. PP-33:22 induced the highest expression of *OCN* at three days. A marked decrease of *OCN* expression was observed at 7 days, the values markedly increased after 14 days. PP-31:31 induced a nine-fold increase of *OCN* expression at 3 and 7 days, less marked at 14 days.

#### 3.6.4. Vascular Differentiation (*CD31* Gene Expression) of MSCs Cultured with 10% Hydrogel Extracts Added to Culture Medium

**Figure 10:**
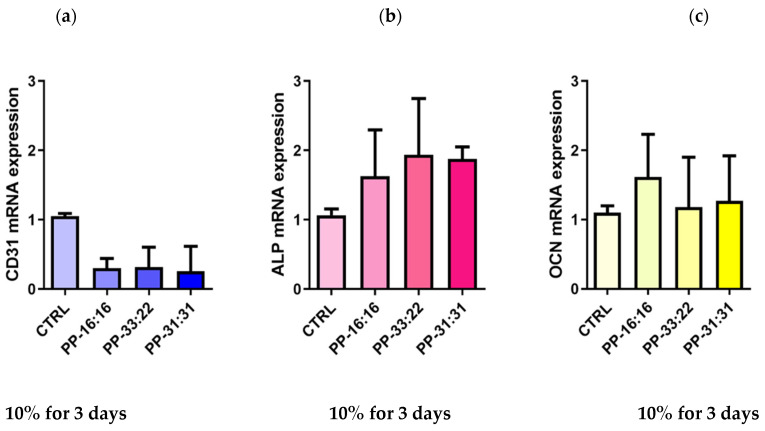
Gene expression of *CD31* (**a**), *ALP* (**b**) and *OCN* (**c**) analyzed by Real Time PCR in MSCs cultured with 10% extracts from mineral-filled hydrogels (PP-16:16, PP-33:22, PP-31:31). Results are reported as fold changes relative to CTRL (control MSCs cells cultured without hydrogels extracts); data are expressed as mean ± SD of three independent experiments. *ALP*: alkaline phosphatase. *OCN*: osteocalcin. Bars indicate the Standard Deviation.

The addition of 10% extracts from each formulation induced *CD31* downregulation in MSCs, as evident in Figure 10a.

#### 3.6.5. Osteogenic Differentiation (*ALP*, *OCN* Gene Expression) of MSCs Cultured with 10% Hydrogel Extracts Added to Culture Medium

The addition of 10% extract from each mineral-filled hydrogel appear to stimulate the osteogenic differentiation of cultured cells, as shown by the mRNA expression of *ALP* and *OCN* (Figure 10b,c).

*ALP* expression was upregulated in all the three experimental formulations (Figure 10b). The extracts from PP-16:16 and PP-31:31 induced a 0.6 and 0.8-fold increase, while the extracts from PP-33:22 induced a 1.9-fold increase.

*OCN* mRNA expression slightly increased in all three formulations (Figure 10c). The addition of 10% extract from PP-16:16 induced a 0.6-fold increase, while the extract from PP-33:22 induced a 0.18-fold increase of *OCN* expression when compared to CTRL (MSC control cells).

## 4. Discussion

The occurrence of bone defects in oral maxilla or mandibula is a frequent clinical condition. The healing of bony deficit, caused by infections (periradicular, abscesses, periimplantitis), periodontitis, cysts, insufficient peri-implant bone, osteoporosis, trauma, tumor excision, and bone necrosis, requires treatments favouring bone regeneration.

There are increasing attention and grants allocated to “green”, i.e., eco-sustainable chemistry and technology. In this context, the use of natural bio-derived compounds (possibly abundant in nature) having high biocompatibility and biological interactivity to design materials for bone regeneration represents a futuristic ecological approach.

Mannuronic/guluronic acid-based polysaccharides can be synthetized from marine seaweed comprised in *Phaeophyceae* class. Commercial polysaccarydes are produced mainly from wild brown algae of *Ascophyllum, Durvillaea, Ecklonia, Laminaria, Lessonia, Macrocystis, Saccharina,* and *Sargassum* genera. Approximately 1500–2000 species of brown algae are present worldwide [48] and are abundant in several world areas, including the Mediterranean, North East Atlantic (North Sea and Baltic Areas), North America, and South East Asia [48].

The coastal invasion of brown algae is a critical worldwide environmental phenomenon reported in Ireland, the Mediterranean Sea, and the Caribbean Sea [49,50]. The harvesting of algae from eutrophic coasts, where the proliferation of invading seaweeds is favoured by water nutrients, represents an attractive green approach adopting a new ecological concept whereby invasive algae are removed from coastal areas for the production of useful algae-based biomaterials (circular chemistry concept).

Mannuronic/guluronic acid-based polysaccharides possess several biological advantages when compared to other synthetic polymers, including hydrophilicity, absence of toxicity, and low immunogenicity [51]. The drawbacks of these polysaccharides, such as low mechanical properties, uncontrolled degradation, and lack of adhesion ligands for mammalian cells, may be avoided/surpassed through their combination with other polymers and/or fillers. This is the strategy that we used to project the grafts of the present study.

Therefore, the design of bio-based composite scaffolds, constituted by different natural polymers having various biological effects favouring bone regeneration processes, represents a very attractive strategy.

Recently, experimental bio-based stiff/solid polymers containing collagen, chitosan, carrageenan, or alginate, in combination with calcium compounds as calcium chloride, calcium phosphate, calcium sulphate, hydroxyapatite, or glass have been conceived and tested [52,53,54].

Scaffolds composed of fibrin, mannuronic/guluronic acid, and calcium phosphate were demonstrated to be non-cytotoxic, biodegradable, biointeractive, and were able to induce pro-osteogenic and angiogenic differentiation on mammalian macrophages [52]. Porous scaffolds made of mannuronic/guluronic acid, carrageenan, and calcium silicate showed fibroblasts biocompatibility and the ability to nucleate apatite [53]. Hydroxyapatite/chitosan and mannuronic/guluronic acid-based scaffolds loaded with antibacterial molecules were biocompatible, released biointeractive calcium ions, and induced the osteogenic differentiation of bone marrow stem cells [54].

The association of mannuronic/guluronic acids with gelatine hydrogel revealed biocompatibility and absence of cytotoxicity for skin tissue engineering [55] or wound healing application [56]. To date, no similar hydrogels have been proposed for clinical application in bone tissue engineering, due to their lack of suitable physical-chemical and biointeractive properties.

In the present study, innovative green mineral-filled hydrogels have been developed starting from bio-derived sources through low-temperature, non-toxic, and low time-consuming procedures. The graft biomaterials have been conceived to be placed in non-load-bearing bone sites or in alveolar bone sites during healing, with the purpose to support bone tissue regeneration. Clinical studies and conventional oral rehabilitation protocols indicate that functional loading can be postponed for several months, waiting for new bone formation [57].

The impact of inorganic fillers addition in hydrogels has been widely reported in literature [58,59,60]. Inorganic mineral fillers, such as calcium phosphates, calcium silicates, and calcium carbonates, provide activating biointeractive signals by releasing biologically active ions [61,62]. Moreover, the fillers anisotropy in the presence of electrical stimulation may be useful to guide the resident cell growth, proliferation, and attachment [58,59,60]. The use of inorganic fillers, such as hydroxyapatite or silicate nanoparticles, may provide additional properties to the soft and flat hydrogel surface, imparting stiffness strength [58,59,60]. The inclusion of specific inorganic fillers (such as silver) in hydrogels may confer antibacterial properties [63,64].

In our study, the rationale for the addition of high percentages of CaSi and DCPD (up to 62% by weight) minerals is therefore related to the need to confer consistence to the hydrogel and to improve the biological properties through biointeractive and biologically active ion-releasing fillers [40,41].

CaSi are widely used in oral surgery and endodontics for their positive bio-interaction with bone tissues (i.e., periapical bone) [21,65,66]. When in contact with body fluids, CaSi can create a microenvironment favorable for apatite nucleation, through the formation of a hydrated silica rich layer, the release of calcium ions, and the increase of local pH [23,29,36]. The leached calcium ions have and epigenetic effect on mineralizing cells [67,68] and silicon possesses angiogenic properties [69,70].

A calcium phosphate component has also been included to provide further positive epigenetic chemical signals to cells involved in regenerative processes of bone. In addition, the association of calcium phosphate compounds and CaSi bioactive material improves and accelerates the apatite nucleation [35]. DCPD provides an additional phosphate source early after contact with watery fluids for its progressive dissolution [33,35]. DCPD is the most soluble calcium orthophosphate salt at pH > 8.2 due to the presence of structural water [71] and converts into apatite after contact with water or fluids.

In the present study, the designed hydrogels can crosslink through divalent ions (i.e calcium ions) rapidly released from CaSi and calcium phosphate. All mineral-filled hydrogels released high calcium ions and showed alkalizing ability during the 28 days test period. The ion release values particularly increased during the first three days and remained stable at all the tested endpoints (seven, 14, and 28 and days).

It has been reported that soluble molecular signals, such as calcium ions, trigger the cascade of cell differentiation into osteoblast lineage [29] and create the conditions for a bone bonding structure (apatite coating layer) useful in bone defects [34]. Released calcium ions specifically modulate osteopontin, *OCN*, and *ALP* in mineralizing cells. The occurrence of alkaline pH increased the activity/growth of osteoblasts and other mineralizing cells [72].

In addition, silicon from CaSi likely provided an additional positive stimulus for mesenchymal stem cell differentiation into vascular lineage [73]. SiOH silanol groups may be exposed by CaSi and increase the nucleation of apatite [74].

Our study analysed the gene expression of markers for osteogenesis (*ALP* and *OCN*) and vascularization (*CD31*) by MSCs cultured in contact with mineral-filled hydrogels and with hydrogels extracts.

*ALP* and *OCN* are markers of initial and late osteogenic differentiation. *ALP* expression increases in cells at alkaline pH during the first stages of mineral phase formation, while *OCN* expression indicates calcified tissue formation. *OCN* binds hydroxyapatite crystals into extracellular calcified matrix [71].

*CD31*, also known as platelet endothelial cell adhesion molecule 1 (PECAM-1), is a sensitive and specific marker for vascular differentiation [75]. In our study, the gene expression of MSCs cultured in contact with mineral-filled hydrogels showed that the addition of increasing percentages of CaSi and DCPD favored the expression of the pro-angiogenic marker *CD31* (Figure 9a), with limited osteogenic marker expression (*ALP* and *OCN*, Figure 9b,c). The expression of this gene was high (over 40-fold increase at 14 days for all the formulations), although non-statistically significant due to the high standard deviation.

Differently, MSCs cultured with hydrogels extracts induced an expression of the osteogenic markers (Figure 10b,c) and not of the angiogenic marker. This could be attributed to the stable calcium ions released by the mineral-filled formulations during the first days immersion, as evident in Table 2.

It should be clarified that the differences in gene expression between the two experimental conditions (MSCs in contact with the mineral-filled hydrogels and MSCs in presence of 10% extracts) can be likely related to a lower concentration of biologically active ions in 10%.

Neoangiogenesis is a necessary prerequisite for bone regeneration, new bone tissue development, and remodeling processes. Oral bone is highly vascularized. An ideal biomaterial should favor vascular endothelial cells attachment, proliferation, and vascular network formation, should allow the nutrients influx and the later mineralizing cell colonization [76,77].

Recent studies [32,78,79] showed the expression of osteogenic and proangiogenic genes by MSCs in contact with CaSi and DCPD in polymer-based scaffolds. However, it has been reported that materials with low mineral filling showed a negligible induction of *OCN* expression by MSCs derived from human periapical cyst after 21 days of culture [32] and a moderate expression of *OCN* from human adipose derived MSCs after 14 days [79]. In addition, a moderate pro-angiogenic stimulus on vascular wall mesenchymal stem cells was originated by materials with low mineral filling [79].

In this study, a reduction of MSCs mortality in presence of hydrogel extracts was observed when higher amounts of mineral fillers were used. Similar and low percentages of dead cells, comparable to the CTRL, was obtained when cells were cultured in the presence of PP-31:31, the formulation with the highest amount of CaSi and DCPD. A previous study on alginate-gelatine scaffold extracts showed that high extracellular calcium ions lead to an improvement of cell viability [80], in agreement with our cell mortality tests. Extracellular calcium ions have an impact on cell behaviour, viability, and proliferation [22,28,29,30,31,33,40,41].

In the present study, the results of cell mortality indicate and confirm that the biologically active ions released from mineral-filled hydrogels with the highest percentage of CaSi-DCPD (i.e., PP:31-31) lead to a reduction of cell mortality and to an improvement of biological properties of the materials (increased gene expression). This effect may also be related to both the hydrophilic behavior of the bio-based hydrogel and to the mineral layer nucleation in the formulation with higher mineral fillers inclusion.

Raman spectroscopy showed that the different phases of mineral-filled hydrogels interact between each other. In particular, conformationally sensitive bands of gelatin (i.e., Amide I and III) underwent shifts and changes in relative intensities, suggesting that the inorganic fillers induced structural rearrangements of the organic phase. Wavenumber shifts were detected also in the bands of the former components.

Raman spectroscopy disclosed B-type carbonated apatite (and calcite) nucleation only on the hydrogel containing the highest number of inorganic fillers, i.e., PP-31:31 (Figure 6 and Figure S4), while such phases were not detected on the other mineral-filled hydrogels. The Raman spectra showed that upon mineral deposition the gelatin component underwent conformational rearrangements towards a more unordered state, as revealed by the wavenumber downshift of Amide I and the strengthening of the 1272 cm^−1^ band. At the same time, the COO^−^ groups belonging to mannuronate/guluronate (and likely also to aspartate and glutamate residues of gelatin) complexed calcium, as revealed by the strengthening of the COO^−^ stretching modes and by the appearance of the band at 1436 cm^−1^ (Figure 6 and Figure 7), assigned to calcium alginate [43].

Raman spectroscopy showed that no calcium ion saturation was attained in the CTRL hydrogel, on the basis of the relatively low shift of the COO^−^ symmetric stretching (from 1414 to 1419 cm^−1^, see Table S1) and breathing modes of the glycosidic ring (from 1096 to 1095 cm^−1^, see Table S1). For calcium ions saturated hydrogels shift by 20 and 10 cm^−1^ have been reported for the former and the latter modes, respectively [44,47].

Our mineral-filled hydrogels showed attractive physical properties when compared with PP-CTRL. High porosity and water sorption likely related to each other and to calcium release were observed, in particular for hydrogels filled with high amounts of CaSi and DCPD (PP-33:22 and PP-31:31) (Table 4).

The formulation with no mineral filler (designed as control) revealed low porosity and water sorption, low ions release and demonstrated complete dissolution after 3–7 days immersion in simulated body fluids.

We underline that high porosity is required for vascularization and neoangiogenesis processes in order to allow the uptake of nutrients and growth factors from peripheral blood vessels followed by cells colonization and proliferation and new blood vessel formation into biomaterial structure [81].

ESEM analysis revealed marked surface modifications of the mineral-filled hydrogels when immersed in HBSS. This may be related to polymer degradation, mineral granules exposition, and apatite nucleation, all indexes of a dynamic modification of the mineral-filled hydrogel structure over time during the contact with body fluid. Indeed, a shift to a more porous surface was observed after 28 days.

All formulations revealed pore sizes over 100 μm after immersion in HBSS, markedly larger than that of fresh hydrogels. PP-16:16, the formulation with a lower percentage of fillers, had very limited pores when compared to PP-31:31 (the formulation with the highest percentage of fillers).

Pore size is important to allow different biological functions. Pores within 20 microns allow cell–extracellular matrix interactions and nutrients/metabolites transport. Pores larger than 100 μm are useful for new vessel formation and new bone formation [82].

The chemical-physical and biological data obtained in the present study support the multifaceted/multimodal action of the designed mineral-filled hydrogels for oral bone defects. Such biomaterials (mainly PP-31:31) can act as vasculogenesis promoters and osteogenesis inducers through the direct stimulation of MSCs by their biointeractive properties and the release of biologically active signals.

## 5. Conclusions

The designed algae-based hydrogels represent a green approach to biomaterial science and bone regenerative medicine.

The incorporation of biointeractive fillers allowed the production of bioactive and porous hydrogels able to create an adequate micro-environment for stem cell activation and with a commitment to vascular and osteoblast lineage, present as interesting graft materials for bone regeneration purposes. Further perspectives include their implantation in an animal model to in vivo investigate the osteoinductive/osteoconductive properties of PP-33:22 and PP-31:31, the formulations containing the highest percentages of fillers.

## Figures and Tables

**Table 1:** Primer sequences used for Real-Time PCR. *ALP* = alkaline phosphatase, *GAPDH* = glyceraldehyde 3-phosphate dehydrogenase, *OCN* = Osteocalcin.

Gene	Forward Sequence	Reverse Sequence
***GAPDH* **	5′–AATGGGCAGCCGTTAGGAAA–3′	5′–AGGAGAAATCGGGCCAGCTA–3′
***CD31* **	5′–CACAGATGAGAACCACGCCT–3′	5′–GGCCCCTCAGAACAACAT–3′
***ALP* **	5′–GACCTCCTCGGAAGACACT–3′	5′–TGAAGGGCTTCTTGTCTGT–3′
***OCN* **	5′–CACCGAGACACCATGAGAGC–3′	5′–CTGCTTGGACACAAAGGCT–3′

**Table 2:** Calcium ions release expressed as ppm (mean ± SD, *n* = 10). Different letters represent statistically significant differences (*p* < 0.05) in the same line (capital letters) or in the same column (small letters).

Materials	0–3 h	3 h–1 Day	1–3 Days	3–7 Days	7–14 Days	14–28 Days	Cumulative
**PP-16:16**	43.73 ± 5.7 aA	60.85 ± 10.89 aB	29.52 ± 5.68 aC	37.53 ± 10.31 aA	31.95 ± 4.65 aC	32.30 ± 13.18 cA	192.17 ± 14.45 a
**PP-33:22**	44.64 ± 11.06 aA	53.078 ± 14.8 aB	54.95 ± 15.07 bB	51.95 ± 9.1 bB	49.01 ± 8.5 bAB	44.15 ± 11.75 bA	253.15 ± 40.64 b
**PP-31:31**	68.19 ± 19.01 bA	71.65 ± 7.6 bA	51.51 ± 5.3 bB	46.72 ± 17.3 bB	32.92 ± 9.01 aC	27.73 ± 7.01 aC	230.54 ± 33.05 b
**PP-CTRL**	1.56 ± 0.28 cA	6.47 ± 0.04 cB	3.58 ± 0.68 cA	2.31 ± 0.94 cA	2.41 ± 1.43 cA	2.46 ± 1.45 cA	17.23 ± 3.9 c

**Table 3:** pH of soaking water (mean ± SD, *n* = 10). Different letters represent statistically significant differences (*p* < 0.05) in the same line (capital letters) or in the same column (small letters).

Materials	3 h	1 Day	3 Days	7 Days	14 Days	28 Days
**PP-16:16**	8.82 ± 0.10 aA	8.86 ± 0.28 aA	8.614 ± 0.34 aA	8.45 ± 0.13 aB	8.33 ± 0.15 aB	8.13 ± 0.16 aC
**PP-33:22**	8.79 ± 0.20 aA	9.23 ± 0.23 bB	8.86 ± 0.12 abA	8.61 ± 0.18 bA	8.49 ± 0.09 aC	8.41 ± 0.23 aC
**PP-31:31**	9.07 ± 0.18 bA	9.27 ± 0.17 bA	8.98 ± 0.18 bA	8.64 ± 0.25 bB	8.46 ± 0.13 aBC	8.32 ± 0.14 aC
**PP-CTRL**	7.74 ± 0.08 cA	7.83 ± 0.17 cA	7.42 ± 0.16 cA	7.75 ± 0.23 aA	7.69 ± 0.16 bA	7.68 ± 0.45 bA

**Table 4:** Porosity, Solubility and Water Sorption (mean ± SD, *n* = 10) after 24 h drying. Different letters represent statistically significant differences (*p* < 0.05) in the same line (capital letters) or in the same column (small letters). WS = Water Sorption, S = Solubility, P = Porosity, Vop = Volume of Open pores, V_ip_ = Volume of impervious portion.

	WS (%)	S (%)	P (%)	V_OP_ (cm^3^)	V_IP_ (cm^3^)
**PP-16:16**	74.45 ± 15.5 a	5.14 ± 1.51 a	46.65 ± 7.45 a	1.554 ± 0.45 a	1.764 ± 0.26 a
**PP-33:22**	147.15 ± 26.2 b	10.12 ± 3.2 b	63.15 ± 9.25 b	2.123 ± 0.38 b	1.265 ± 0.24 b
**PP-31:31**	224.29 ± 26.56 c	21.31 ± 9.4 c	76.23 ± 12.65 b	2.514 ± 0.15 b	0.912 ± 0.22 b
**PP-CTRL**	54.45 ± 16.0 a	5.26 ± 3.94 a	36.47 ± 0.91 c	1.21 ± 0.15 a	2.17 ± 0.15 c

**Table 5:** Radiopacity (mm Al, *n* = 3).

**PP-16:16**	<1
**PP-33:22**	<1
**PP-31:31**	<1
**PP-CTRL**	<1

**Table 6:** Initial setting time (hours, *n* = 3).

**PP-16:16**	96
**PP-33:22**	96
**PP-31:31**	96
**PP-CTRL**	Unset after 144 h

## Data Availability

Not applicable.

## Supplementary Materials

The following are available online at www.mdpi.com/article/10.3390/nano11123439/s1, Figure S1. Average FT-Raman spectra of fresh CTRL hydrogel, gelatin and sodium alginate powders. Figure S2. Average FT-Raman spectra of fresh PP-31:31, fresh CTRL hydrogel, DCDP and CaSi powders. Figure S3. Average FT-Raman spectra of fresh PP-16:16, PP-33:22 and PP-31:31 in the Amide I range. Figure S4. Micro-Raman spectra recorded on two different points (p1 and p2) of the PP-31:31 scaffold aged in HBSS for 28 days. Figure S5. Average FT-Raman spectra of fresh PP-16:16 and after ageing for 28 days in HBSS. Figure S6. Average FT-Raman spectra of fresh PP-33:22 and after ageing for 28 days in HBSS. Table S1. 1800–300 cm^−1^ wavenumbers (cm^−1^) of the main Raman bands of fresh CTRL hydrogel, gelatin and poly(sodium D-mannuronate-co-L-guluronate) powders. Table S2. 1800-100 cm^−1^ wavenumbers (cm^−1^) of the main Raman bands of fresh PP-31:31, fresh CTRL hydrogel, DCDP and CaSi powders. Table S3. 1800–250 cm^−1^ wavenumbers (cm^−1^) of the main Raman bands of fresh PP-31:31 and after ageing in HBSS for 28 days.

## References

[1] Oryan A., Alidadi S., Moshiri A., Maffulli N. (2014). Bone regenerative medicine: Classic options, novel strategies, and future directions. J. Orthop. Surg. Res..

[2] Bai X., Gao M., Syed S., Zhuang J., Xu X., Zhang X.-Q. (2018). Bioactive hydrogels for bone regeneration. Bioact. Mater..

[3] Jahangirian H., Lemraski E.G., Rafiee-Moghaddam R., Webster T.J. (2018). A review of using green chemistry methods for biomaterials in tissue engineering. Int. J. Nanomed..

[4] Roi A., Ardelean L.C., Roi C.I., Boia E.-R., Boia S., Rusu L.-C. (2019). Oral Bone Tissue Engineering: Advanced Biomaterials for Cell Adhesion, Proliferation and Differentiation. Materials.

[5] Gerecht-Nir S., Cohen S., Ziskind A., Itskovitz-Eldor J. (2004). Three-dimensional porous alginate scaffolds provide a conducive environment for generation of well-vascularized embryoid bodies from human embryonic stem cells. Biotechnol. Bioeng..

[6] Gong Y., Han G.T., Zhang Y.M., Zhang J.F., Jiang W., Tao X.W., Gao S.C. (2016). Preparation of alginate membrane for tissue engineering. J. Polym. Eng..

[7] Degli Esposti M., Chiellini F., Bondioli F., Morselli D., Fabbri P. (2019). Highly porous PHB-based bioactive scaffolds for bone tissue engineering by in situ synthesis of hydroxyapatite. Mater. Sci. Eng. C Mater. Biol. Appl..

[8] Saeed M., Beigi-Boroujeni S., Rajabi S., Ashteiani G.R., Dolatfarahi M., Özcan M. (2021). A simple, green chemistry technology for fabrication of tissue-engineered scaffolds based on mussel-inspired 3D centrifugal spun. Mater. Sci. Eng. C.

[9] Garner J., Park K., Ramawat K., Mérillon J.M. (2015). Chemically Modified Natural Polysaccharides to Form Gels. Polysaccharides.

[10] Moussa D.G., Aparicio C. (2018). Present and future of tissue engineering scaffolds for dentin-pulp complex regeneration. J. Tissue Eng. Regen. Med..

[11] Dalheim M.Ø., Vanacker J., Najmi M.A., Aachmann F.L., Strand B.L., Christensen B.E. (2016). Efficient functionalization of alginate biomaterials. Biomaterials.

[12] Shaheen T., Montaser A., Li S. (2019). Effect of cellulose nanocrystals on scaffolds comprising chitosan, alginate and hydroxyapatite for bone tissue engineering. Int. J. Biol. Macromol..

[13] Drury J.L., Mooney D.J. (2003). Hydrogels for tissue engineering: Scaffold design variables and applications. Biomaterials.

[14] Ganguly S., Das P., Itzhaki E., Hadad E., Gedanken A., Margel S. (2020). Microwave-Synthesized Polysaccharide-Derived Carbon Dots as Therapeutic Cargoes and Toughening Agents for Elastomeric Gels. ACS Appl. Mater. Interfaces.

[15] Sharma S., Srivastava D., Grover S., Sharma V. (2014). Biomaterials in tooth tissue engineering: A review. J. Clin. Diagn. Res..

[16] Dubey N., Ferreira J.A., Daghrery A., Aytac Z., Malda J., Bhaduri S.B., Bottino M.C. (2020). Highly tunable bioactive fiber-reinforced hydrogel for guided bone regeneration. Acta Biomater..

[17] Yan J., Miao Y., Tan H., Zhou T., Ling Z., Chen Y., Xing X., Hu X. (2016). Injectable alginate/hydroxyapatite gel scaffold combined with gelatin microspheres for drug delivery and bone tissue engineering. Mater. Sci. Eng. C.

[18] Liu P., Shen H., Zhi Y., Si J., Shi J., Guo L., Shen S.G. (2019). 3D bioprinting and in vitro study of bilayered membranous construct with human cells-laden alginate/gelatin composite hydrogels. Colloids Surf. B Biointerf..

[19] Lopa S., Madry H. (2014). Bioinspired Scaffolds for Osteochondral Regeneration. Tissue Eng. Part A.

[20] Joddar B., Garcia E., Casas A., Stewart C. (2016). Development of functionalized multi-walled carbon-nanotube-based alginate hydrogels for enabling biomimetic technologies. Sci. Rep..

[21] Prati C., Gandolfi M.G. (2015). Calcium silicate bioactive cements: Biological perspectives and clinical applications. Dent. Mater..

[22] Gandolfi M.G., Taddei P., Siboni F., Modena E., Ciapetti G., Prati C. (2011). Development of the foremost light-curable calcium-silicate MTA cement as root-end in oral surgery. Chemical–physical properties, bioactivity and biological behavior. Dent. Mater..

[23] Gandolfi M.G., Taddei P., Modena E., Siboni F., Prati C. (2013). Biointeractivity-related versus chemi/physisorption-related apatite precursor-forming ability of current root end filling materials. J. Biomed. Mater. Res. Part B Appl. Biomater..

[24] Gandolfi M.G., Siboni F., Botero T., Bossù M., Riccitiello F., Prati C. (2015). Calcium Silicate and Calcium Hydroxide Materials for Pulp Capping: Biointeractivity, Porosity, Solubility and Bioactivity of Current Formulations. J. Appl. Biomater. Funct. Mater..

[25] Taddei P., Tinti A., Gandolfi M.G., Rossi P., Prati C. (2009). Vibrational study on the bioactivity of Portland cement-based materials for endodontic use. J. Mol. Struct..

[26] Gandolfi M.G., Taddei P., Siboni F., Modena E., Ginebra M.P., Prati C. (2011). Fluoride-containing nanoporous calcium-silicate MTA cements for endodontics and oral surgery: Early fluorapatite formation in a phosphate-containing solution. Int. Endod. J..

[27] Zamparini F., Siboni F., Prati C., Taddei P., Gandolfi M.G. (2019). Properties of calcium silicate-monobasic calcium phosphate materials for endodontics containing tantalum pentoxide and zirconium oxide. Clin. Oral Investig..

[28] Gandolfi M.G., Ciapetti G., Perut F., Taddei P., Modena E., Rossi P.L., Prati C. (2010). Biomimetic calcium-silicate cements aged in simulated body solutions. Osteoblast response and analyses of apatite coating. J. Appl. Biomater. Biomech..

[29] Gandolfi M.G., Ciapetti G., Taddei P., Perut F., Tinti A., Cardoso M.V., Van Meerbeek B., Prati C. (2010). Apatite formation on bioactive calcium-silicate cements for dentistry affects surface topography and human marrow stromal cells proliferation. Dent. Mater..

[30] Gandolfi M.G., Shah S.N., Feng R., Prati C., Akintoye S.O. (2011). Biomimetic Calcium-Silicate Cements Support Differentiation of Human Orofacial Mesenchymal Stem Cells. J. Endod..

[31] Hakki S.S., Bozkurt B.S., Ozcopur B., Gandolfi M.G., Prati C., Belli S. (2012). The response of cementoblasts to calcium phosphate resin-based and calcium silicate-based commercial sealers. Int. Endod. J..

[32] Tatullo M., Spagnuolo G., Codispoti B., Zamparini F., Zhang A., Degli Esposti M., Aparicio C., Rengo C., Nuzzolese M., Manzoli L. (2019). PLA-Based Mineral-Doped Scaffolds Seeded with Human Periapical Cyst-Derived MSCs: A Promising Tool for Regenerative Healing in Dentistry. Materials.

[33] Gandolfi M.G., Spagnuolo G., Siboni F., Procino A., Rivieccio V., Pelliccioni G.A., Prati C., Rengo S. (2015). Calcium silicate/calcium phosphate biphasic cements for vital pulp therapy: Chemical-physical properties and human pulp cells response. Clin. Oral Investig..

[34] Gandolfi M., Iezzi G., Piattelli A., Prati C., Scarano A. (2017). Osteoinductive potential and bone-bonding ability of ProRoot MTA, MTA Plus and Biodentine in rabbit intramedullary model: Microchemical characterization and histological analysis. Dent. Mater..

[35] Gandolfi M.G., Taddei P., Tinti A., Dorigo E.D.S., Prati C. (2011). Alpha-TCP improves the apatite-formation ability of calcium-silicate hydraulic cement soaked in phosphate solutions. Mater. Sci. Eng. C.

[36] Gandolfi M.G., Taddei P., Tinti A., Dorigo E.D.S., Rossi P.L., Prati C. (2009). Kinetics of apatite formation on a calcium-silicate cement for root-end filling during ageing in physiological-like phosphate solutions. Clin. Oral Investig..

[37] David L.C., Hewlett P.C. (1998). The constitution and specification of Portland cements. Leas’s Chemistry of Cement and Concrete.

[38] Siboni F., Taddei P., Prati C., Gandolfi M.G. (2017). Properties of NeoMTA Plus and MTA Plus cements for endodontics. Int. Endod. J..

[39] Camilleri J., Gandolfi M.G. (2010). Evaluation of the radiopacity of calcium silicate cements containing different radiopacifiers. Int. Endod. J..

[40] Gandolfi M.G., Zamparini F., Degli Esposti M., Chiellini F., Aparicio C., Fava F., Fabbri P., Taddei P., Prati C. (2018). Polylactic acid-based porous scaffolds doped with calcium silicate and dicalcium phosphate dihydrate designed for biomedical application. Mater. Sci. Eng. C.

[41] Gandolfi M.G., Zamparini F., Degli Esposti M., Chiellini F., Fava F., Fabbri P., Taddei P., Prati C. (2019). Highly porous polycaprolactone scaffolds doped with calcium silicate and dicalcium phosphate dihydrate designed for bone regeneration. Mater. Sci. Eng. C.

[42] Stoppel W.L., White J.C., Horava S.D., Henry A.C., Roberts S.C., Bhatia S.R. (2014). Terminal sterilization of alginate hydrogels: Efficacy and impact on mechanical properties. J. Biomed. Mater. Res. Part B Appl. Biomater..

[43] Valente S., Alviano F., Ciavarella C., Buzzi M., Ricci F., Tazzari P.L., Pagliaro P., Pasquinelli G. (2014). Human cadaver multipotent stromal/stem cells isolated from arteries stored in liquid nitrogen for 5 years. Stem Cell Res. Ther..

[44] Schmid T., Messmer A., Yeo B.S., Zhang W., Zenobi R. (2008). Towards chemical analysis of nanostructures in biofilms II: Tip-enhanced Raman spectroscopy of alginates. Anal. Bioanal. Chem..

[45] Andersen F.A., Brecevic L., Beuter G., Dell’Amico D.B., Calderazzo F., Bjerrum N.J., Underhill A.E. (1991). Infrared Spectra of Amorphous and Crystalline Calcium Carbonate. Acta Chem. Scand..

[46] Nelson D.G., Featherstone J.D. (1982). Preparation, analysis, and characterization of carbonated apatites. Calcif. Tissue Int..

[47] Pielesz A., Klimczak M., Bak K. (2008). Raman spectroscopy and WAXS method as a tool for analyzing ion-exchange properties of alginate hydrogels. Int. J. Biol. Macromol..

[48] Beata Łabowska M., Michalak I., Detyna J. (2019). Methods of extraction, physicochemical properties of alginates and their applications in biomedical field—A review. Open Chem..

[49] Fletcher R., Farrell P. (1998). Introduced brown algae in the North East Atlantic, with particular respect toUndaria pinnatifida (Harvey) suringar. Helgol. Meeresunters.

[50] Engelen A., Serebyakova A., Ang P., Britton-Simmon K., Mineur F., Pedersen M.F., Arenas F., Fernández C., Steen S., Svenson R. (2015). Circumglobal Invasion by the Brown Seaweed Sargassum muticum. Ocean Mar. Biol..

[51] Lee K.Y., Mooney D.J. (2012). Alginate: Properties and biomedical applications. Prog. Polym. Sci..

[52] Kohli N., Sharma V., Orera A., Sawadkar P., Owji N., Frost O.G., Bailey R.J., Snow M., Knowles J.C., Blunn G.W. (2021). Pro-angiogenic and osteogenic composite scaffolds of fibrin, alginate and calcium phosphate for bone tissue engineering. J. Tissue Eng..

[53] Sathain A., Monvisade P., Siriphannon P. (2021). Bioactive alginate/carrageenan/calcium silicate porous scaffolds for bone tissue engineering. Mater. Today Commun..

[54] Liu D., Liu Z., Zou J., Li L., Sui X., Wang B., Yang N., Wang B. (2021). Synthesis and Characterization of a Hydroxyapatite-Sodium Alginate-Chitosan Scaffold for Bone Reg eneration. Front. Mater..

[55] Solovieva E.V., Fedotov A.Y., E Mamonov V., Komlev V.S., A Panteleyev A. (2017). Fibrinogen-modified sodium alginate as a scaffold material for skin tissue engineering. Biomed. Mater..

[56] Afjoul H., Shamloo A., Kamali A. (2020). Freeze-gelled alginate/gelatin scaffolds for wound healing applications: An in vitro, in vivo study. Mater. Sci. Eng. C.

[57] Gallucci G.O., Hamilton A., Zhou W., Buser D., Chen S. (2018). Implant placement and loading protocols in partially edentulous patients: A systematic review. Clin. Oral Implant. Res..

[58] Zhao F., Yao D., Guo R., Deng L., Dong A., Zhang J. (2015). Composites of Polymer Hydrogels and Nanoparticulate Systems for Biomedical and Pharmaceutical Applications. Nanomaterials.

[59] Utech S., Boccaccini A.R. (2016). A review of hydrogel-based composites for biomedical applications: Enhancement of hydrogel properties by addition of rigid inorganic fillers. J. Mater. Sci..

[60] Moini N., Jahandideh A., Anderson G. (2019). Inorganic Nanocomposite Hydrogels: Present Knowledge and Future Challenge. Sustainable Polymer Composites and Nanocomposites.

[61] Saveleva M., Prikhozhdenko E., Gorin D., Skirtach A.G., Yashchenok A., Parakhonskiy B. (2020). Polycaprolactone-Based, Porous CaCO_3_ and Ag Nanoparticle Modified Scaffolds as a SERS Platform With Molecule-Specific Adsorption. Front. Chem..

[62] Lishchynskyi O., Stetsyshyn Y., Raczkowska J., Awsiuk K., Orzechowska B., Abalymov A., Skirtach A., Bernasik A., Nastyshyn S., Budkowski A. (2021). Fabrication and Impact of Fouling-Reducing Temperature-Responsive POEGMA Coatings with Embedded CaCO_3_ Nanoparticles on Different Cell Lines. Materials.

[63] Kaniewska K., Karbarz M., Katz E. (2020). Nanocomposite hydrogel films and coatings—Features and applications. Appl. Mater. Today.

[64] Nastyshyn S., Raczkowska J., Stetsyshyn Y., Orzechowska B., Bernasik A., Shymborska Y., Brzychczy-Włoch M., Gosiewski T., Lishchynskyi O., Ohar H. (2020). Non-cytotoxic, temperature-responsive and antibacterial POEGMA based nanocomposite coatings with silver nanoparticles. RSC Adv..

[65] Zamparini F., Pelliccioni G.A., Spinelli A., Gissi D.B., Gandolfi M.G., Prati C. (2021). Root canal treatment of compromised teeth as alternative treatment for patients receiving bisphosphonates: 60-month results of a prospective clinical study. Int. Endod. J..

[66] Chybowski E.A., Glickman G.N., Patel Y., Fleury A., Solomon E., He J. (2018). Clinical Outcome of Non-Surgical Root Canal Treatment Using a Single-cone Technique with Endosequence Bioceramic Sealer: A Retrospective Analysis. J. Endod..

[67] Rashid F., Shiba H., Mizuno N. (2003). The effect of extracellular calcium ion on gene expression of bone-related proteins in human pulp cells. J. Endod..

[68] Matsumoto S., Hayashi M., Suzuki Y., Suzuki N., Maeno M., Ogiso B. (2013). Calcium Ions Released from Mineral Trioxide Aggregate Convert the Differentiation Pathway of C2C12 Cells into Osteoblast Lineage. J. Endod..

[69] Day R.M. (2005). Bioactive Glass Stimulates the Secretion of Angiogenic Growth Factors and Angiogenesis in Vitro. Tissue Eng..

[70] Zhai W., Lu H., Chen L., Lin X., Huang Y., Dai K., Naoki K., Chen G., Chang J. (2012). Silicate bioceramics induce angiogenesis during bone regeneration. Acta Biomater..

[71] Takagi S., Chow L., Ishikawa K. (1998). Formation of hydroxyapatite in new calcium phosphate cements. Biomaterials.

[72] Okabe T., Sakamoto M., Takeuchi H., Matsushima K. (2006). Effects of pH on Mineralization Ability of Human Dental Pulp Cells. J. Endod..

[73] Sun J., Wei L., Liu X., Li J., Li B., Wang G., Meng F. (2009). Influences of ionic dissolution products of dicalcium silicate coating on osteoblastic proliferation, differentiation and gene expression. Acta Biomater..

[74] Gandolfi M.G., Taddei P., Tinti A., Prati C. (2010). Apatite forming ability of ProRoot MTA. Int. Endod. J..

[75] Franceschi R.T., Iyer B.S. (2009). Relationship between collagen synthesis and expression of the osteoblast phenotype in MC3T3-E1 cells. J. Bone Miner. Res..

[76] Vanchinathan V., Mizramani N., Kantipudi R., Schwartz E.J., Sundram U.N. (2015). The Vascular Marker *CD31* Also Highlights Histiocytes and Histiocyte-Like Cells within Cutaneous Tumors. Am. J. Clin. Pathol..

[77] Bramfeld H., Sabra G., Centis V., Vermette P. (2010). Scaffold Vascularization: A Challenge for Three-Dimensional Tissue Engineering. Curr. Med. Chem..

[78] Gandolfi M.G., Gardin C., Zamparini F., Ferroni L., Degli Esposti M., Parchi G., Ercan B., Manzoli L., Fava F., Fabbri P. (2020). Mineral-Doped Poly(L-lactide) Acid Scaffolds Enriched with Exosomes Improve Osteogenic Commitment of Human Adipose-Derived Mesenchymal Stem Cells. Nanomaterials.

[79] Forni M., Bernardini C., Zamparini F., Zannoni A., Salaroli R., Ventrella D., Parchi G., Degli Esposti M., Polimeni A., Fabbri P. (2020). Vascular Wall–Mesenchymal Stem Cells Differentiation on 3D Biodegradable Highly Porous CaSi-DCPD Doped Poly (α-hydroxy) Acids Scaffolds for Bone Regeneration. Nanomaterials.

[80] Yu H., Zhang X., Song W., Pan T., Wang H., Ning T., Wei Q., Xu H.H., Wu B., Ma D. (2019). Effects of 3-dimensional Bioprinting Alginate/Gelatin Hydrogel Scaffold Extract on Proliferation and Differentiation of Human Dental Pulp Stem Cells. J. Endod..

[81] Karageorgiou V., Kaplan D. (2005). Porosity of 3D biomaterial scaffolds and osteogenesis. Biomaterials.

[82] Perez R.A., Mestres G. (2016). Role of pore size and morphology in musculo-skeletal tissue regeneration. Mater. Sci. Eng. C.

