# Supplementary material for: Green Hydrogels Composed of Sodium Mannuronate/Guluronate, Gelatin and Biointeractive Calcium Silicates/Dicalcium Phosphate Dihydrate Designed for Oral Bone Defects Regeneration
Source: Nanomaterials (Basel). 2021 Dec 18;11(12):3439. doi: 10.3390/nano11123439 (PMC8706657; doi:10.3390/nano11123439)
Supplement: Supplementary file 1 [file nanomaterials-11-03439-s001.zip › nanomaterials-1481233-supplementary.pdf]

Supplementary Material

Green Hydrogels Composed of Sodium Mannuronate/Guluronate, Gelatin and Biointeractive Calcium Silicates/Dicalcium Phosphate Dihydrate Designed for Oral Bone Defects Regeneration

Maria Giovanna Gandolfi [1,*], Fausto Zamparini [1,2], Sabrina Valente [3], Greta Parchi [1], Gianandrea Pasquinelli [3,4], Paola Taddei [5] and Carlo Prati [2]

[1] Laboratory of Green Biomaterials and Oral Pathology, School of Dentistry, DIBINEM, University of Bologna, Bologna, 40125, Italy; fausto.zamparini2@unibo.it (F.Z.); greta.parchi@studio.unibo.it (G.P.)
[2] Endodontic Clinical Section, School of Dentistry, DIBINEM, University of Bologna, Bologna, 40125, Italy; carlo.prati@unibo.it
[3] Department of Experimental, Diagnostic and Specialty Medicine, DIMES, University of Bologna, Bologna, 40138, Italy; sabrina.valente2@unibo.it (S.V.); gianandr.pasquinelli@unibo.it (G.P.)
[4] Subcellular Nephro-Vascular Diagnostic Program, Pathology Unit, IRCCS Azienda Ospedaliero-Universitaria di Bologna, Bologna, 40138, Italy
[5] Biochemistry Unit, DIBINEM, University of Bologna, Bologna, 40126, Italy; paola.taddei@unibo.it
* Correspondence: mgiovanna.gandolfi@unibo.it

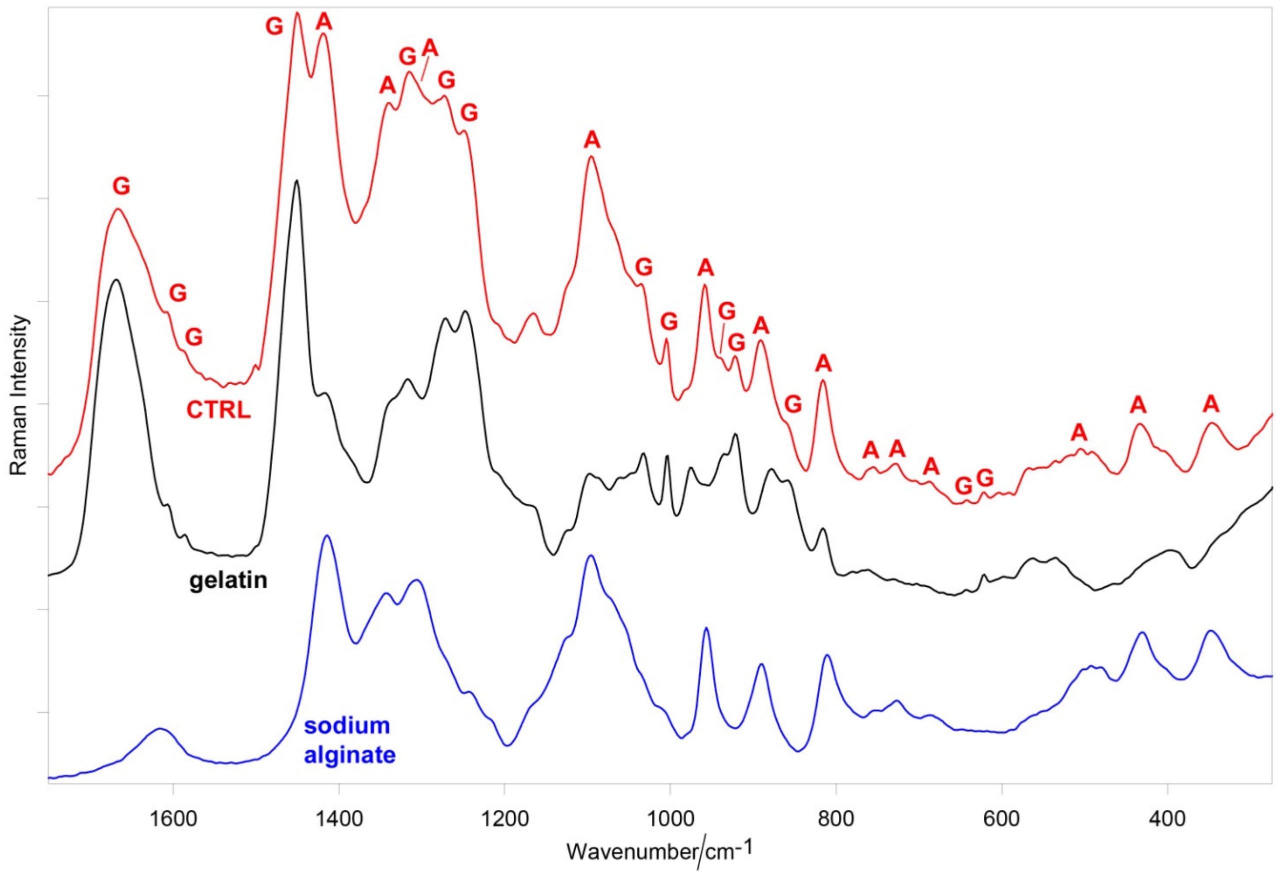

Figure S1. Average FT-Raman spectra of fresh CTRL hydrogel, gelatin and sodium alginate powders. Band wavenumbers are reported in Table S1, Supplementary Material. The main bands prevalently assignable to gelatin (G) and poly(sodium D-mannuronate-co-L-guluronate) (A) are indicated. When both components contribute to a band, the main contribution is indicated.

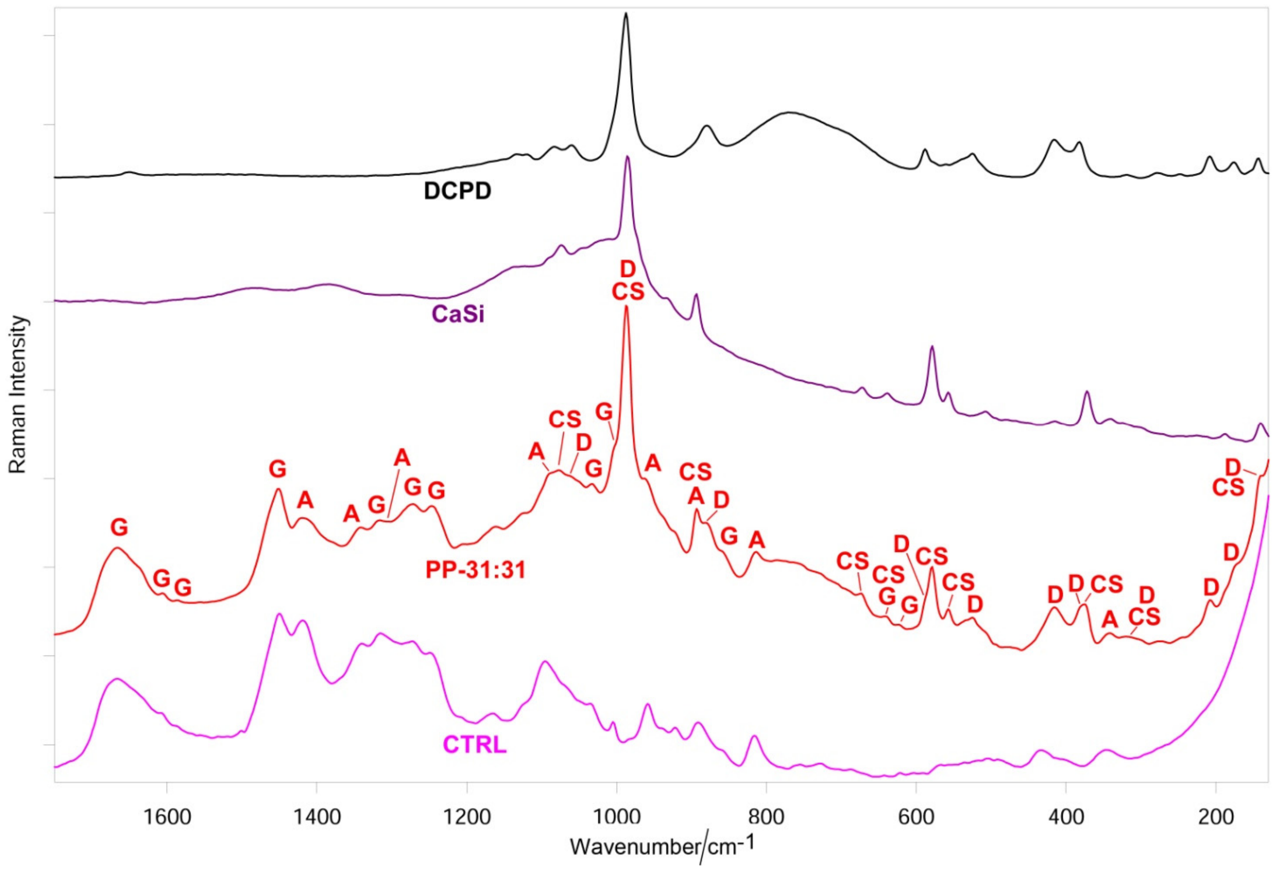

Figure S2. Average FT-Raman spectra of fresh PP-31:31, fresh CTRL hydrogel, DCDP and CaSi powders. Band wavenumbers are reported in Table S2, Supplementary Material. The bands prevalently assignable to gelatin (G), poly(sodium D-mannuronate-co-L-guluronate) (A), DCPD (D) and CaSi hydraulic cement (CS) are indicated.

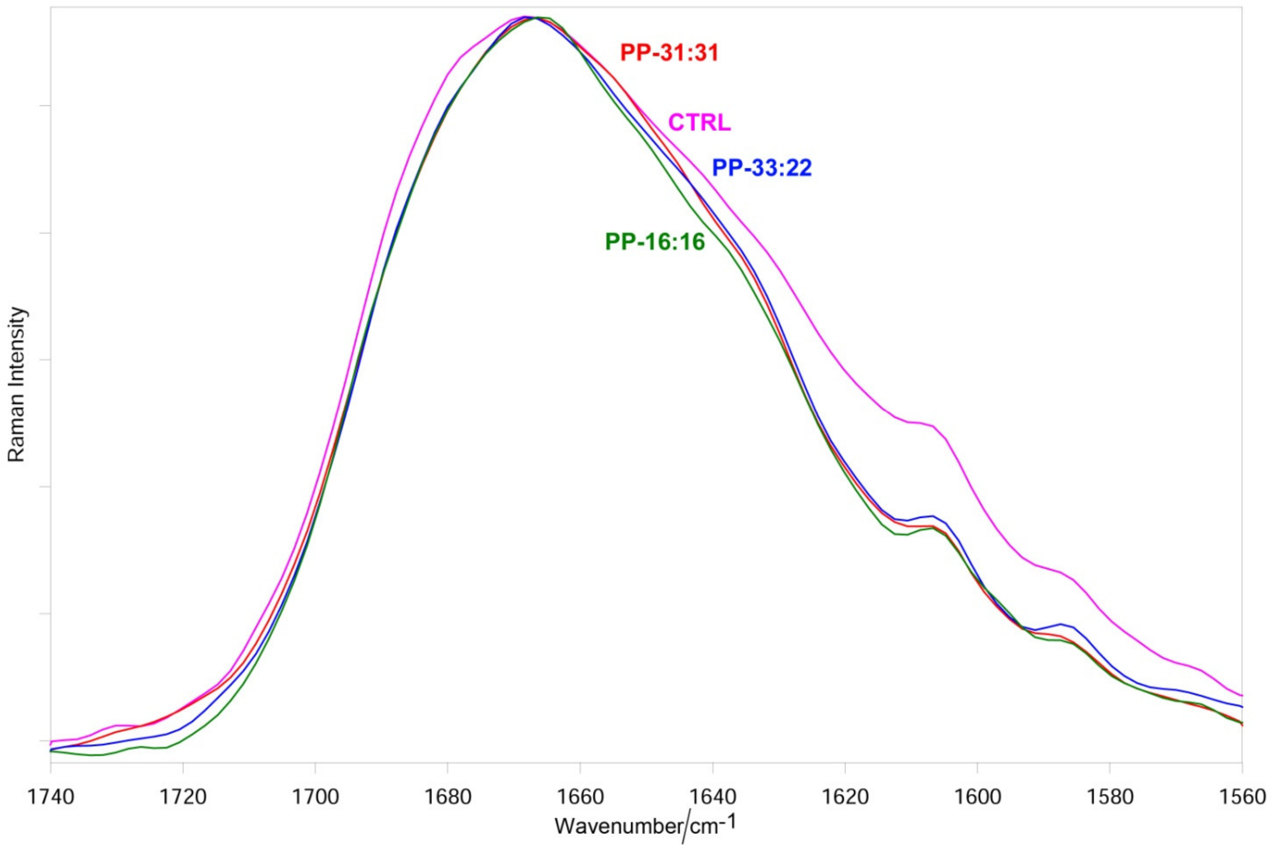

Figure S3. Average FT-Raman spectra of fresh PP-16:16, PP-33:22 and PP-31:31 in the Amide I range.

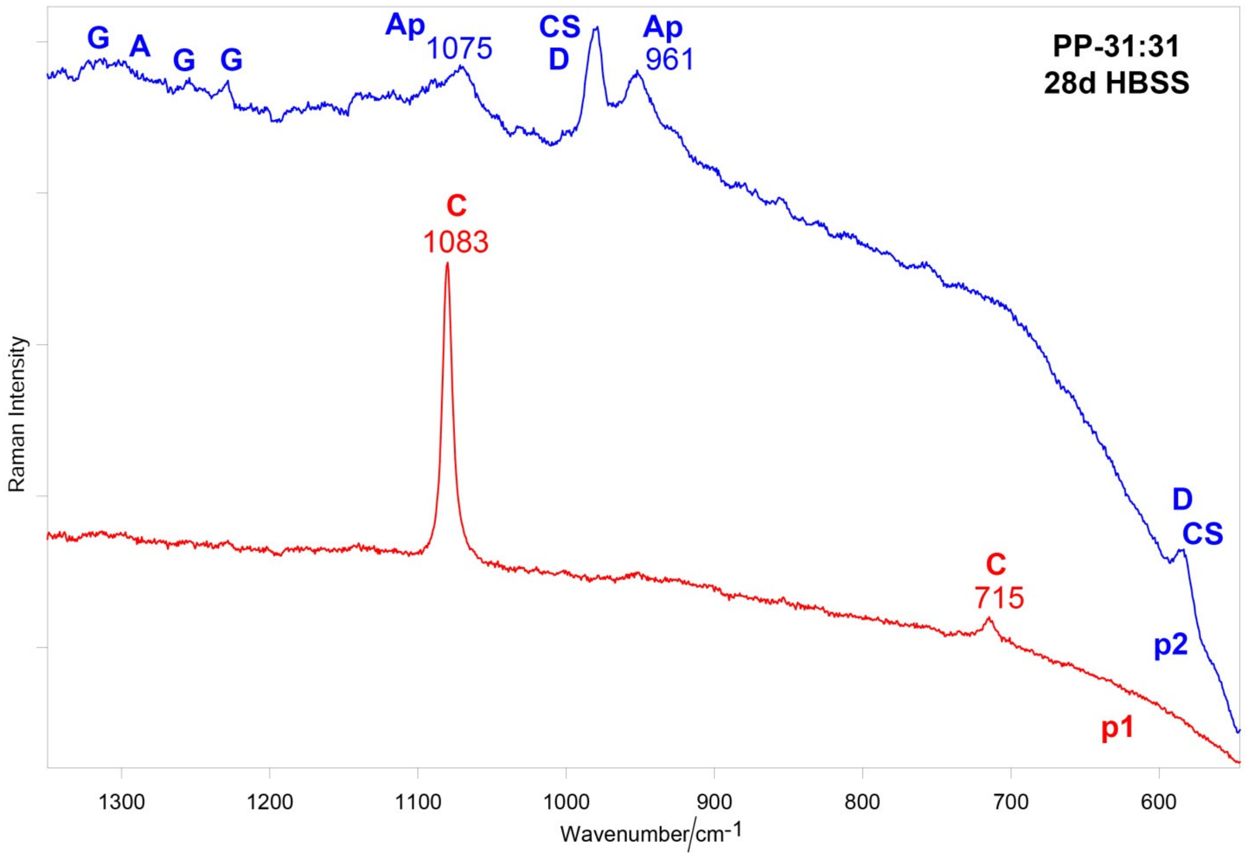

Figure S4. Micro-Raman spectra recorded on two different points (p1 and p2) of the PP-31:31 scaffold aged in HBSS for 28 days. The bands prevalently assignable to gelatin (G), poly(sodium D-mannuronate-co-L-guluronate) (A), DCPD (D), CaSi hydraulic cement (CS), calcite (C) and B-type carbonated apatite (Ap) are indicated.

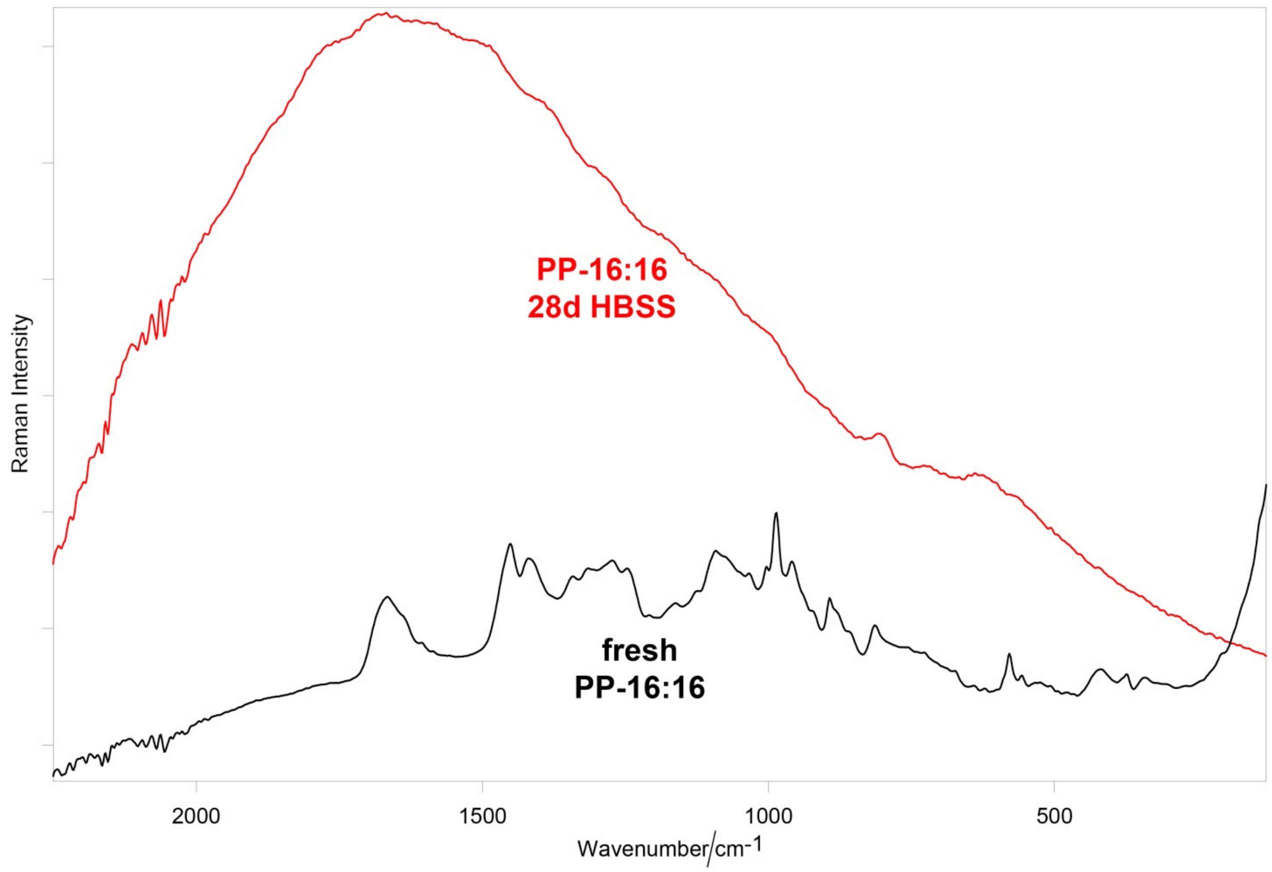

Figure S5. Average FT-Raman spectra of fresh PP-16:16 and after ageing for 28 days in HBSS.

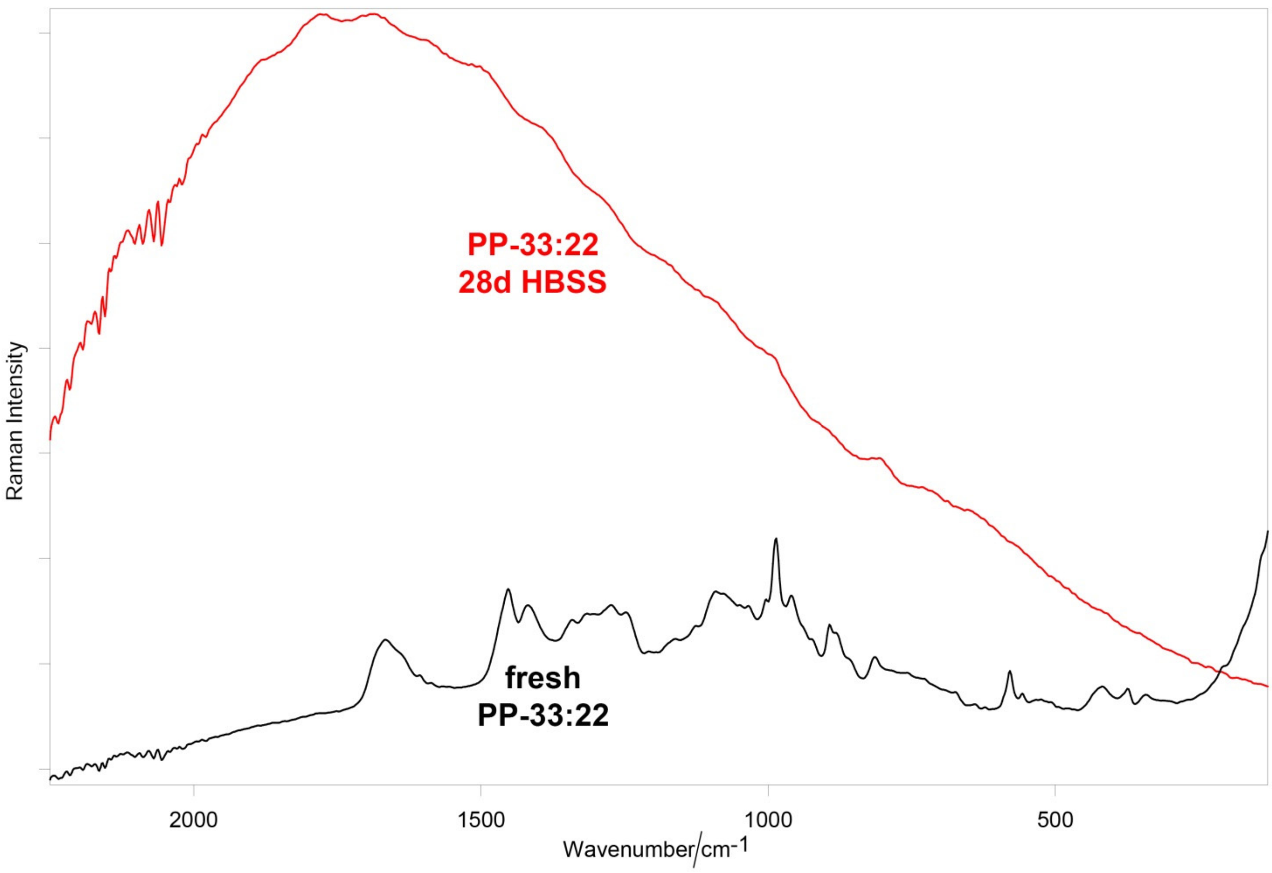

Figure S6. Average FT-Raman spectra of fresh PP-33:22 and after ageing for 28 days in HBSS.

Table S1. 1800–300 cm^{-1} wavenumbers (cm^{-1}) of the main Raman bands of fresh CTRL hydrogel, gelatin and poly(sodium D-mannuronate-co-L-guluronate) powders.

CTRL hydrogel	Gelatin powder	Poly(sodium D-mannuronate-co-L-guluronate) powder
1667	1669	
		1616
1608	1607	
1588	1586	
1450	1451	
1419	1417	1414
1340	1337 (shoulder)	1343
1315	1317	
1308 (shoulder)		1306
1273	1272	
1249	1248	1243
1095	1098	1096
1035	1033	
1005	1004	
958		957
942	937	
922	922	
891		890
862	878-858	
816	816	811
755	762	
728		727
688		687
643	643	
622	622	
505		492
434		431
347		348

Table S2. 1800–100 cm^{-1} wavenumbers (cm^{-1}) of the main Raman bands of fresh PP-31:31, fresh CTRL hydrogel, DCDP and CaSi powders.

PP-31:31	CTRL hydrogel	DCPD	CaSi
1666	1667		
1606	1608		
1587	1588		
1451	1450		
1421	1419		
1341	1340		
1316	1315		
1272	1273		
1247	1249		
1090	1095		
1077			1074
1061		1060	
1033	1035		
	1005		
987		987	985
962	958		
923	922		
893	891		894
880		879	
861	862		
814	816		
674			672
641	643		639
624	622		
588 (shoulder)		588	
579			579
557			557
525		525	
416		416	415
380 (shoulder)		382	
375			372

342	347		341
320		319	323
275		276	
245		248	
208		208	
188		176	
140		143	140

Table S3. 1800–250 cm^{-1} wavenumbers (cm^{-1}) of the main Raman bands of fresh PP-31:31 and after ageing in HBSS for 28 days.

Fresh PP-31:31	PP-31:31 in HBSS	Assignments
1666	1658	
1606	1606	
1587	1587	
1451	1450 (shoulder)	
	1436 (shoulder)	
1421	1421	
1341	1339	
1316	1315	
1272	1272	
1247	1247	
1090		
	1087	calcite
1077		
	1076	B-type carbonated apatite
1061		
1033		
987	985	
962 shoulder		
	959 strong	B-type carbonated apatite
923		
893	890	
880		

861	860	
814	810	
	714	calcite
674		
641	639	
624		
588 (shoulder)	585 (shoulder)	
579	579	
557	558	
525	508	
416	418	
380 (shoulder)	380 (shoulder)	
375	373	
342	339	
320		
	281	calcite
